# Radical scavenger competition of alizarin and curcumin: a mechanistic DFT study on antioxidant activity

**DOI:** 10.1007/s00894-021-04778-1

**Published:** 2021-05-13

**Authors:** Malek Sadatsharifi, Mihály Purgel

**Affiliations:** 1 University of Debrecen, Egyetem tér 1, Debrecen, H-4032 Hungary; 2 Department of Physical Chemistry, University of Debrecen, Egyetem tér 1, Debrecen, H-4032 Hungary

**Keywords:** Epoxide, Mechanism, Peroxo bond, Cleavage, Degradation, Autoxidation

## Abstract

In vivo hydroxyl, peroxyl, and superoxide free radicals caused by oxidative stress can be toxic to molecules that are essential for the human body. However, there are natural compounds that can decrease the amount of these harmful species. In this work, we are focusing on two well-known compounds, alizarin (red) and curcumin, to study their interactions with these small radicals for a comparison between a rigid and a flexible structure. We made a mechanistic study and found the major and minor degradation products of curcumin as well as the autoxidation products of it based on a wide range of literature. We found several more favored pathways than those that were previously proposed. On the contrary, for degradation/oxidation of alizarin, only a few proposed mechanisms can be found which were performed in specific conditions. Our calculations predicted some favored rearrangements for the alizarin by peroxyl and superoxide radicals.

**Supplementary Information:**

The online version contains supplementary material available at 10.1007/s00894-021-04778-1.

**Figure:**
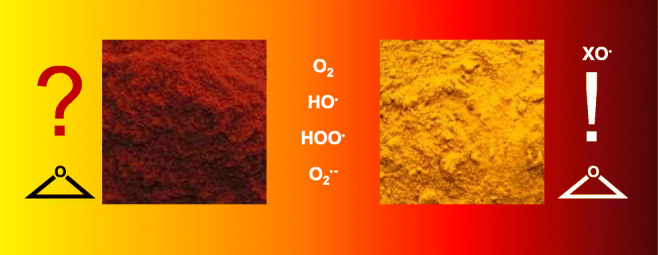
Interaction of alizarin red and bright yellow curcumin with small radicals like hydroxyl, peroxyl, and superoxide radicals, such as the reaction between curcumin radicals and oxygen molecule, results in different species like epoxides or another kind of radical forms. The stability of epoxides is different in the case of rigid and flexible structures.

## Introduction

**Scheme 1:**
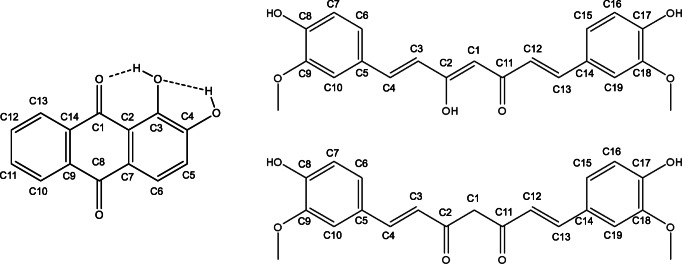
Structures and numbering of the most stable form of alizarin and curcumin tautomers

During the last decade, much research has been conducted examining the biological effects of free radicals [1–6] and antioxidants [7–9]. Free radicals are dangerous for living cells, and therefore compounds that can scavenge them are an interesting subject for experimentation [10]. Diseases can be caused by radicals, but nowadays researchers are focusing more on natural compounds, mainly phenolics, as potential therapeutic agents to block the metabolic disorders (diabetes, cancer, etc.) of free radicals [11–13]. In the present work, we are focusing on alizarin and curcumin which are biologically active [14, 15] and on their interactions with potentially toxic molecules such as hydroxyl, peroxyl, and superoxide radicals [16–18]. These are mainly defined as reduced oxygen agents but in general; they can be categorized as reactive oxygen species (ROS). These are highly reactive regarding membrane lipids, protein, and DNA, and in general, they can damage cellular components and are probably responsible for stress injuries and conditions as well [19, 20]. Both alizarin and curcumin are the subjects of substantial literature in many different areas. Their usage as antioxidants has been actively explored during the last few decades; however, mechanisms and detailed effects are still not fully understood [21, 22]. Alizarin (1,2-dihydroxy-9,10-anthraquinone) can be isolated from the roots of the common madder (*Rubia tinctorum L.*) [23]. It is used as a Japanese food colorant, and its carcinogenic activity was proven in rats [24]. Clinical applications (bio-labeling, etc.) are typical research topics involving alizarin (AR) compounds as well [24, 25]. AR is a well-known pH indicator due to the changes in its color in terms of (de)protonation of the –OH group at the C2 position (see Scheme 1). It is a quinone, and quinones are known as radical competitors that can stabilize radical forms [26]. Benzoquinone can uptake hydrogen atoms, but it is unable to lose hydrogen from its aromatic ring. Alizarin, however, has an extended ability to form a radical not only by uptaking hydrogen atoms but also by losing hydrogen from hydroxyl groups. Curcumin {CM; (1E,6E)-1,7-bis(4-hydroxy-3-methoxyphenyl)hepta-1,6-diene-3,5-dione} exists in two tautomeric forms (see Scheme 1) and has been isolated from *Curcuma longa* and found to be a promising natural antioxidant; therefore, it is widely investigated by researchers. It is a compound of turmeric that has been used for centuries as a natural and traditional medicine in Asia for various diseases (liver ailments, parasitic infections, skin diseases, inflammations, and flu symptoms). In recent years, curcumin has been studied as a therapeutic agent and as a cell protector against Alzheimer’s disease [27–30]. Moreover, the anticancer effect of curcumin has been shown as a pro-oxidant by generating reactive oxidant species (ROS) [31], and its anticarcinogenic effect concerning concentration dependence has been proven as well [32].

**Scheme 2:**
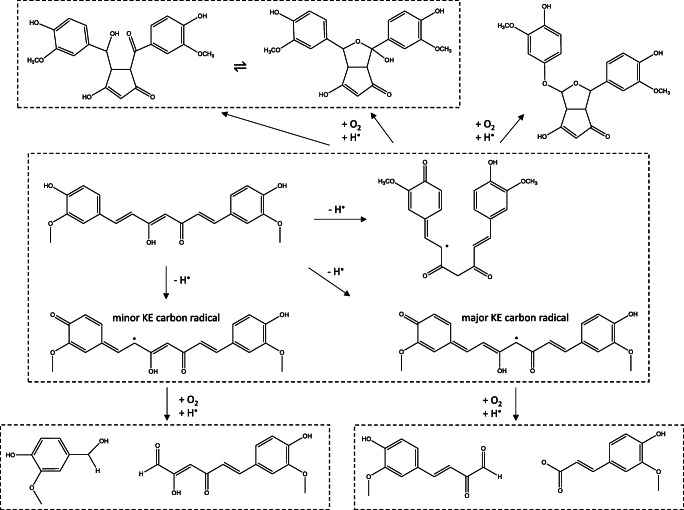
Proposed autoxidation and degradation processes by experiments

Besides, the decomposition of curcumin has been studied in physiological conditions by Wang et al., and *trans*-6-(4′-hydroxy-3′-methoxyphenyl)-2,4-dioxo-5-hexenal was found as major while *o*-vanillin, ferulic acid, and feruloyl methane were identified as minor degradation products [33]. Masuda et al. found, however, that degradation processes in nonpolar medium went through radical formation, in which oxygen molecule coordinated to the carbon minor and carbon major radical species. From those adducts, cyclizations were proposed resulting in four centered CCOO radical species, and depending on the position of the cyclic unit, vanillin and *trans*-6-(4′-hydroxy-3′-methoxyphenyl)-2,4-dioxo-5-hexenal or ferulic acid and feruloyl methane were identified as products [34, 35]. Schneider and co-workers found that oxygen caused an autoxidative transformation of curcumin including several structures which formation was time-dependent, where hemiacetalcyclopentadione, hydroxyketocyclopentadione, and bicyclopentadione were the main products [36–38]. They found that between 20 and 45 min, spiroepoxide and vinylether were also dominant species. In contrast to Masuda’s and Wang’s results, they proposed a mechanism in which a diketo isomer radical form played an important role in the beginning due to the flexibility of the radical species. This flexible structure allowed the formation of several cyclo and bicyclo species. The most important species of the proposed degradation and autoxidation process can be seen in Scheme 2.

Curcumin’s antioxidant properties have been studied in detail. The active site of curcumin has been defined differently: Tonnesen et al. found that keto-enol formation is responsible for the radical scavenger ability [39] while others claimed that the phenolic group is an active site of scavenging [40, 41]. Jovanovic et al. found that C–H cleavage results in active radical species [42]. Due to all these facts, curcumin and alizarin are relevant agents similar to other natural compounds for both the pharmacological and food industry [43]. Both molecules have an extended delocalized electronic system which is a potential partner to stabilize small radicals like hydroxyl, peroxyl, or superoxide radicals, i.e., to form stable radicals with them. Several papers focus strongly on the type of radical formation. These types can be defined as hydrogen atom transfer (HAT), single-electron transfer followed by proton transfer (SET-PT), and sequential proton loss electron transfer (SPLET) [44]. In most computational studies about radical scavengers and their reactions to free radicals, the type of radical formation has been discussed; however, we have found that there is a lack of knowledge about the adduct formation of these well-investigated compounds [45–48]. It is worth mentioning that the degradation of alizarin has been also studied experimentally but only a few mechanistic studies could be found under specific conditions resulting in completely decomposed structures [49, 50].

Adducts formed by antioxidants are discussed as follows: interaction and adduct formation in alizarin (AR) and curcumin (CM) with small radicals such as hydroxo radical (^•^OH), peroxyl radical (^•^OOH), and superoxide radical (O_2_^•–^)―see the equations below. We studied the rearrangement of the adducts for finding more stable forms, i.e., products in association with the experimental results. On the one hand, we wanted to study the possible rearrangements of alizarin by ROS. On the other hand, we wanted to see whether the proposed mechanisms involving radical forms of curcumin and oxygen molecule are preferred or if another mechanism such as curcumin and small radical interactions can compete with them.

1 $$ \mathrm{AR}/\mathrm{CM}+{\mathrm{OH}}^{\bullet}\to \mathrm{AR}/{\mathrm{CM}}^{\bullet }+{\mathrm{H}}_2\mathrm{O} $$

2 $$ \mathrm{AR}/\mathrm{CM} +^{\bullet}\mathrm{OH}\to \mathrm{AR}/\mathrm{CM}-{\mathrm{OH}}^{\bullet } $$

3 $$ \mathrm{AR}/\mathrm{CM} +^{\bullet}\mathrm{OOH}\to \mathrm{AR}/{\mathrm{CM}}^{\bullet }+{\mathrm{H}}_2{\mathrm{O}}_2 $$

4 $$ \mathrm{AR}/\mathrm{CM}+{\mathrm{OOH}}^{\bullet}\to \mathrm{AR}/\mathrm{CM}-{\mathrm{OOH}}^{\bullet } $$

5 $$ \mathrm{AR}+{{\mathrm{O}}_2}^{\bullet \hbox{--}}\to {\mathrm{AR}}^{\bullet \hbox{--} }+{}^{\bullet}\mathrm{OOH} $$

6 $$ \mathrm{AR}+{{\mathrm{O}}_2}^{\bullet \hbox{--}}\to \mathrm{AR}-{{\mathrm{O}}_2}^{\bullet \hbox{--} } $$

7 $$ \mathrm{AR}+{{\mathrm{O}}_2}^{\bullet \hbox{--}}\to {\mathrm{AR}}^{\bullet \hbox{--} }+{\mathrm{O}}_2 $$

## Computational methods 

Density functional theory (DFT) calculations have been carried out using the Gaussian09 software package [51]. Geometry optimizations have been performed with M06-2X functional [52] and TZVP basis sets [53]. Frequency calculations have been done at the theoretical level of geometry optimization. Relative free energies (ΔG) are reported at 298.15 K and atmospheric pressure. The solvent (water) effect accounted for the universal SMD (solvation model based on solute electron density) model [54]. All stationary points―minima and transition states (TS)―were proven by frequency analysis where minima had all positive frequencies, and TSs had one imaginary frequency related to the actual movement of the reaction coordinate. All energies are given in kcal mol^−1^. In this work, we referred energies to an interacted AR/CM-small radical unit, but we did some comparisons to the separated molecular model to evaluate the effects of our models; see details in the Supplementary Material.

## Results and discussion

### Alizarin radical adduct formation

Alizarin has 14 carbon atoms which are potential partners for radical species, although there are differences among them. Despite the planar structure of AR, the chemical environment around carbon atoms can play an important role since AR can form hydrogen bonds with small radicals. Therefore, in all cases, we tried to build starting geometries where the number of H-bonds is maximal.

We found that H^•^ loss from –OH on C4 position (AR^C4•^) is preferred over –OH of C3 (AR^C3•^) by 1.4 kcal mol^−1^ which is in good agreement with previous results [46]. AR^C4•^ has a planar structure while AR^C3•^ has a slightly bent. We optimized the radicals derived by hydrogen loss from the aromatic ring and found that those are 24.5–26.7 kcal mol^−1^ higher in energy than AR^C4•^. It is not surprising but predicts less relevance for dimerization reactions [55]. We found that in some orientations, there was a barrierless hydrogen transfer to the ^•^OH which formed water and AR^C4•^; however, a transition state was found in the case of the coordination to the aromatic plane (adduct formation). The reactant of this elementary step was set as an AR···^•^OH reference in which the ^•^OH was 268 pm far from the C4 atom of AR. It is worth mentioning that by M06, we could find both transition states of water and adduct formations. Coordination to the aromatic ring had a lower activation barrier (see Table S6) but both water and adduct formation were extremely favored thermodynamically. By M062X/TZVP/SMD, ΔG was −22.6 kcal mol^−1^ for C4-adduct, −19.6 kcal mol^−1^ for C6-adduct, and − 32.2 kcal mol^−1^ for water formation. Note that ^•^OH can interact with AR in different positions, i.e., can get close to AR from many directions, e.g., perpendicular to the aromatic ring; therefore, an adduct formation can be also an option for a radical scavenger. It is worth mentioning that under specific conditions, the complete degradation of alizarin could be detected, and the role of hydroxyl radical was taken into account in the proposed mechanisms [49, 50].

**Scheme 3:**
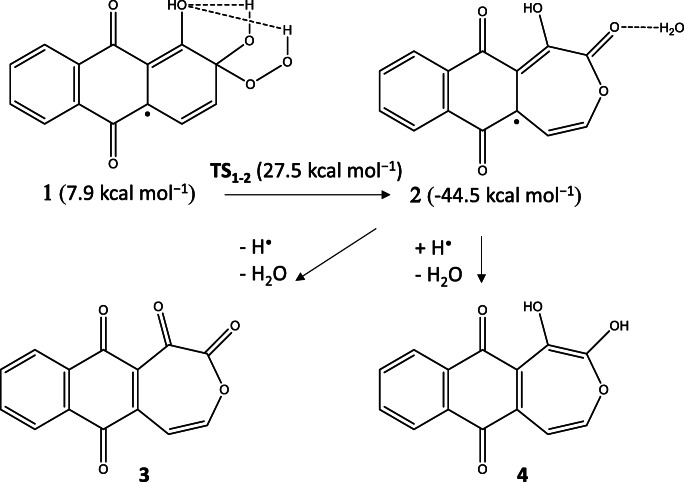
Proposed mechanism for the alizarin–OOH radical systems found by M062X/TZVP/SMD

In the case of ^•^OOH, both transition state and the related reactants could be found by M062X and were shown the kinetic preference of an adduct formation: Δ^‡^G was 19.9 kcal mol^−1^ for C4-adduct, 22.6 kcal mol^−1^ for C6-adduct, and 26.6 kcal mol^−1^ for H_2_O_2_ formation. Thermodynamically, the H_2_O_2_ formation was found as a preferred reaction: ΔG was 7.9 kcal mol^−1^ for C4-adduct (form **1**), 9.6 kcal mol^−1^ for C6-adduct, and 1.4 kcal mol^−1^ for H_2_O_2_ formation. The adducts, however, were found only as intermediates from which a preroxo bond cleavage could occur. From the C4-adduct the activation barrier was 27.5 kcal mol^−1^ (**TS**_**1–2**_) and resulted in an extremely stable heptacyclo radical species together with a water molecule (**2**; −44.5 kcal mol^−1^) after the C–C cleavage of the temporarily formed epoxide group (see Scheme 3). It is worth mentioning that the epoxide form could be found by both M06 functional and ab initio MP2 methods. The peroxo bond cleavage starting from C6-adduct was significantly higher (39.0 kcal mol^−1^) resulting in an epoxide form (−24.2 kcal mol^−1^). The radical form **2** was re-optimized as a singlet state by adding and omitting one hydrogen atom. We found that the formation of the diketo derivative (**3**) is energetically less favored than the formation of the reduced form (**4**) of the quinone (ΔG = −68.4 kcal mol^−1^ vs. -75.1 kcal mol^−1^). The optimized structures of the intermediate can be seen in Fig. S1.

**Scheme 4:**
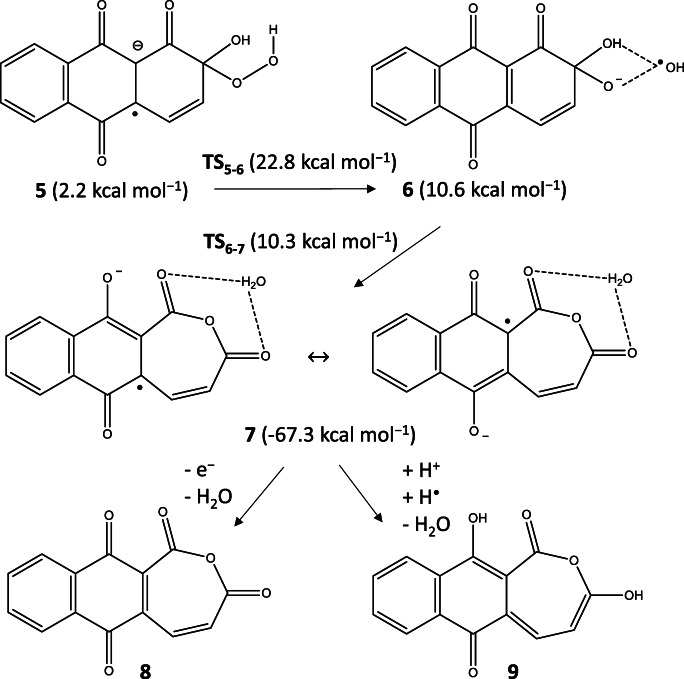
Proposed mechanism for the alizarin–OO^−^ radical systems found by M062X/TZVP

We also studied the interaction between alizarin and superoxide radical. No TS was found for the proton transfer from AR to superoxide radical, but the one in which the proton was closer to the O_2_^•–^ had slightly lower energy than the reference one (ΔG = −0.8 kcal mol^−1^). We found that the superoxide “over” the aromatic ring had higher energy due to the lack of hydrogen bond (4.9 kcal mol^−1^). This form was optimized as a quartet state, and it was found that O–O decreased from 130 pm to 119 pm predicting the oxygen molecule formation and had somewhat higher energy than the doublet state of the same orientation (6.3 kcal mol^−1^). The peroxyl radical coordinated to the C4 atom of the deprotonated alizarin by 18.2 kcal mol^−1^ barrier resulted in a stable adduct (**5**; 2.2 kcal mol^−1^) that was lower than the coordination of the O_2_^•–^ to the AR (24.7 kcal mol^−1^). From **5** which is the deprotonated form of C4 adduct, the peroxo bond cleavage went through a lower barrier (**TS**_**5–6**_) than in the case of **TS**_**1–2**_ by 4.7 kcal mol^−1^ resulting in **6** (10.6 kcal mol^−1^) in which the negative charge was mostly located on the remaining –O^−^ group. The C4 atom of **6** had also a–OH group, from which a proton transfer could occur almost spontaneously (**TS**_**6–7**_), and resulted in another stable heptacyclo derivative (**7**). Note that **6** had lower electronic energy than **TS**_**6–7**_ by 1.6 kcal mol^−1^. We optimized **7** as a neutral species (**8**) and a reduced form (**9**) and was found that both the oxo heptacyclo derivative was more stable than **3** by 14.5 kcal mol^−1^ and the reduced form was also more stable than **4** by 24.1 kcal mol^−1^. The oxidation products **3** and **8** are ether derivatives of tropolones which widely occur in nature [56] and can be synthesized from polyhydroxy benzene derivatives [57]. In the case of crocipodin and thujaplicin synthesis, the oxidizing agent was hydrogen peroxide. The structures of the stationary points can be seen in Scheme 4. Note that the formation was the same by MP2 and M06 methods skipping an epoxide species.

### Curcumin radical formation

Curcumin has two tautomer forms: a diketo (DK) and a keto-enol (KE) isomer (see Scheme 2); the latter one is more stable by 2.4 kcal mol^−1^. It is in good agreement with the literature since enol form can be the dominant (up to 95%) isomer depending on the solvent [58, 59]. Our calculated energy difference predicts only 1–2% of keto form due to the Boltzmann distribution.

**Scheme 5:**
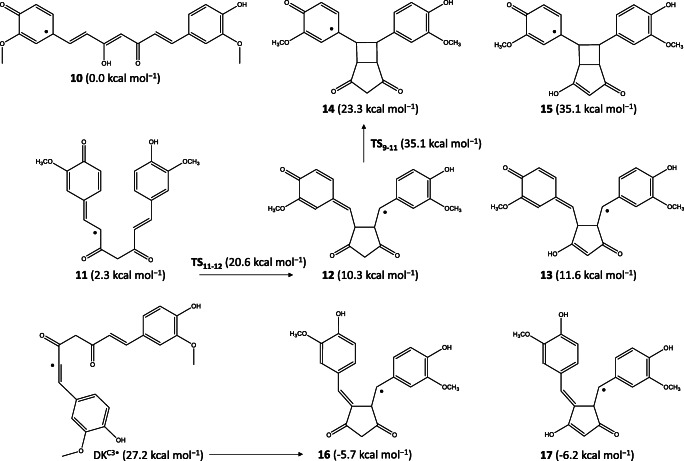
Relevant radical forms of the pre-autoxidation process. Energies are calculated by M062X/TZVP/SMD

Since DK has an sp^3^ carbon in the central position, only 18 carbon atoms are a potential partner for adduct formation, while in the case of KE the central C1 carbon atom can play an important role. We found that hydrogen atom loss from the –OH group of the KE isomer at the C8 position (KE^C8•^; **10** in Scheme 5) was slightly more stable than the other side (KE^C17•^; 0.5 kcal mol^−1^). The radical proposed by Jovanovic (DK^C1•^) [42] was, however, unfavored (12.0 kcal mol^−1^), while other radicals formed by H-loss from C3, C4, C12, and C13 atoms had very high relative energies (over 25 kcal mol^−1^). The interaction of these radicals with oxygen molecule was also studied which were previously suggested in the literature [34–38] as well as the adduct formation by peroxyl and superoxide radicals; see later.

Gordon, Schneider et al. proposed that DK^C8•^ which is a *para*-quinone methide derivative can play an important role in the autoxidation of curcumin [35]. We studied the proposed mechanism, and firstly we did calculations to find the most stable forms, i.e., the most preferred pathway for the pre-autoxidation process (Scheme 5), in which there is no oxygen molecule. We found that the bent conformer of DK^C8•^ (**11**) proposed by Schneider et al. was slightly more stable than the non-bent conformer (2.3 vs. 3.1 kcal mol^−1^) due to the stacking interaction between the aromatic rings. Among the optimized forms **11** and **13**, the non-radical form of **15** was published in their work. We found, however; that the diketo form **12** was slightly but the DK isomer **14** was strongly preferred in contrast to the KE radicals **11** and **13**.

Furthermore, we found other very stable cyclo-intermediates (**16** and **17**), which can be formed by an H-transfer from the pentagonal cyclo-ring to the keto group of the quinonoid ring because the direct formation of **16** from a high-energy species (DK^C3•^; 27.2 kcal mol^−1^) is less favored than the formation of **12** (**TS**_**11–12**_; 20.6 kcal mol^−1^). The main reason for the stabilization of **16** and **17** could be based on the huge structural difference. While **12** and **13** had an unfavored bent quinone after the cyclization step, then in **16** and **17**, the aromatic rings were almost perpendicular to each other resulting in no strain for the C=C bond (see Fig. S2).

### Autoxidation of curcumin

**Scheme 6:**
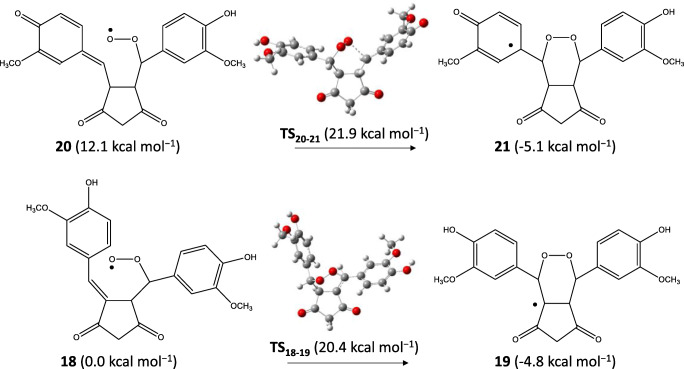
The first step of the autoxidation process. Energies are calculated by M062X/TZVP/SMD

The autoxidation process was studied starting from **12**, **13**, **16**, and **17**, and it was found that DK isomers were preferred to form adducts by an oxygen molecule (see Scheme 6). It can be seen that the preference of **16** over **12** is balanced during the peroxo bridge formation; **18** was much more stable than **20**, but the TSs and the intermediates **19** and **21** had almost the same energies.

Since experimentally the KE isomers were identified, we represent the further rearrangement of the KE radicals; however, it is worth mentioning that in general, DK radicals are preferred by 2–4 kcal mol^−1^ (M06 results), but the final non-radical products are more stable as KE isomer. For example, from **22** (the KE isomer of **21**), the transition state of the epoxide formation had higher energy (**TS**_**22–23**_; 24.9 kcal mol^−1^) than for DK isomer (22.6 kcal mol^−1^); however, the DK analog of **23** was an exception which had slightly higher energy (−11.2 kcal mol^−1^) than the KE radical species **23**. A hydrogen transfer from the ring of **23** to the –O^•^ group resulted in a very stable form (**24**) from which epoxide opening could occur in two ways.

**Scheme 7:**
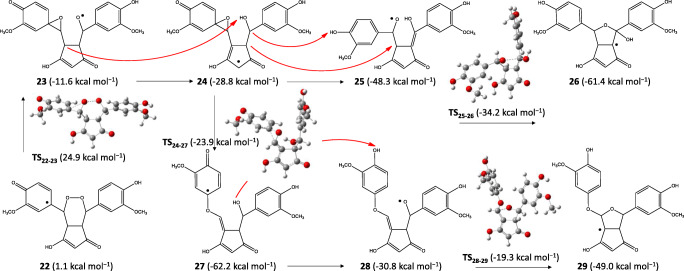
Scheme of the autoxidation process of the KE isomer involving the epoxide intermediate. Energies are calculated by M062X/TZVP/SMD

One of them could be a multistep process including two hydrogen transfers and causing the epoxide unit opening and the quinone → phenolic ring transition (**24** → **25**). From **25**, a bicyclopentadione radical (**26**) was formed by the coordination of the –O^•^ group through a not high barrier (see Scheme 7). By uptaking of a hydrogen atom, the hydroxyketocyclopentadione product derived from **25** was slightly more stable than the hemiacetalcyclopentadione derived from **26** (ΔG = 0.6 kcal mol^−1^) predicting the equilibrium as was discussed by Gordon, Schneider et al. [37]. They proposed that both products could be formed from the proposed but non-isolated endoperoxide intermediate (derived from **21** and **22** by uptaking a hydrogen atom). We found that both endoperoxide isomers had extremely high energy referred to hydroxyketocyclopentadione (KE isomer was at 72.2 kcal mol^−1^; DK isomer was at 67.4 kcal mol^−1^). Schneider et al. declared that the opening of the endoperoxide moiety could occur by a Kornblum–DeLaMare rearrangement [60]. We found that the deprotonation of C–H of the endoperoxide’s pentadione ring by an OH^−^ was both kinetically and thermodynamically preferred (Δ^‡^G = 4.1 kcal mol^−1^; ΔG_r_ = −10.3 kcal mol^−1^), but not surprisingly, the deprotonated species had higher energy than the one which was deprotonated on the –OH of the endoperoxide’s DK isomer (ΔG = 12.9 kcal mol^−1^). The main problem was, however; that calculations showed an unfavored formation for the DK isomer of the endoperoxide intermediate by the reaction of **21** + water molecule (Δ^‡^G = 37.9 kcal mol^−1^; ΔG_r_ = 33.3 kcal mol^−1^). Therefore, we concluded that the formation of hydroxyketocyclopentadione and hemiacetalcyclopentadione could be through radical species (**23** → **26**; see Scheme 7). Note that the activation barrier for the elementary step of the epoxide formation from DK isomer **21** was 27.7 kcal mol^−1^ (TS from **21** had the energy of 22.6 kcal mol^−1^) but from the less stable KE isomer **22**, the elementary step had 23.8 kcal mol^−1^ barrier (see Scheme 7).

The non-radical spiroepoxide derived from **23**/**24** (39.4 kcal mol^−1^) as well as the isolated products of the other branch of autoxidation; the vinylethercyclopentadione product derived from **27**/**28** (14.3 kcal mol^−1^) and the bicyclopentadione derived from **29** (8.7 kcal mol^−1^) had high energy referred to hydroxyketocyclopentadione product. Experimentally, however, the bicyclopentadione was found as the major product. This contradiction can be simply explained by that from the epoxide radical **24**, the CC cleavage of the epoxide unit could occur through a low barrier (**TS**_**24–27**_) resulting in the very stable vinylethercyclopentadione radical (**27**) while the **24** → **25** → **26** pathway had a more complicated rearrangement including outer sphere hydrogen transfers which could be kinetically controlled. However, from **27**, a much less favored radical species (**28**) was found after a hydrogen atom transfer from the –OH group to the oxygen atom of the quinonic ring which seems to be much easier than the H transfers predicted for the **24** → **25** transition including H transfers from and to the carbon atoms of the cyclopentadione ring. The resting –O^•^ group of **28** could also coordinate to sp^2^ carbon atom forming the bicyclopentadione radical (**29**). We found that bicyclopentadione had *cis* configuration to the cyclopentadione ring as was found experimentally; the *trans* isomer had 20.7 kcal mol^−1^ higher energy than the *cis* isomer. Despite that the orientation of the –OH group was good relative to the cyclopentadione ring in form **27**, the formation of the *trans* radical species was also unfavored. Spiroepoxide and vinylethercyclopentadione were also detected experimentally which can be due to the unfavored long-term rearrangements of outer-sphere hydrogen transfers in the case of **24** → **25** and **27** → **28**.

Gordon et al. proposed that the spiroepoxide intermediate had an oxygen exchange found by ^18^O NMR. Due to the very stable **26** form, we suggest that the oxygen exchange occurs after the epoxide ring opens through a low barrier; therefore, **26** is a kind of thermodynamic cage during the process.

### Degradation of curcumin by small radicals

We found that the Jovanovic-type radical (DK^C1•^) formed an adduct with oxygen molecule which had higher energy (15.1 kcal mol^−1^) than the cyclopentadione DK forms **18** and **20** in Scheme 6. The peroxo bond cleavage starting from it had very high activation energy (51.9 kcal mol^−1^) due to the concerted step of the C–C and O–O cleavages resulting in the very stable product of ferulic acetate and an aldehyde radical derivative of feruloyl methane (−49.0 kcal mol^−1^).

The O_2_ adducts of minor and major KE carbon radicals defined by Matsuda et al. had much higher energies (29.6 and 31.7 kcal mol^−1^) than other previously mentioned O_2_ adduct species. We studied the cyclization from these species and found that the activation barriers were very high (50.2 and 39.3 kcal mol^−1^) which showed a kinetic preference for the major product between the two unfavored pathways. The OO-cyclic products having four-membered rings were also unstable (21.3 and 24.1 kcal mol^−1^). Masuda et al. concluded that these species formed in a non-polar medium; therefore, we made a single point in vacuo calculations for **20**, **21**, and the minima forms proposed by Masuda. It was found that **20** had higher energy by 4.5 kcal mol^−1^, while Masuda’s species had somewhat lower energies (2–4 kcal mol^−1^) referred to **21**, but **20** remained more stable than Matsuda’s species in vacuo.

**Scheme 8:**
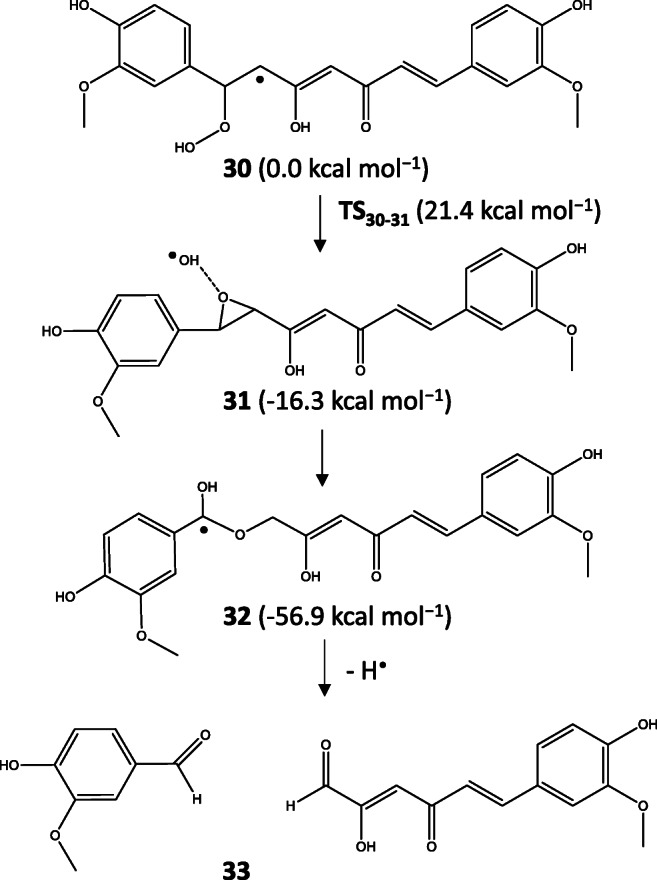
Proposed mechanism starting from the C4 adduct of the curcumin + peroxyl radical system resulting in vanillin and 6-(4′-hydroxy-3′-methoxyphenyl)-2,4-dioxo-5-hexenal

Due to the high energies of the O_2_ adducts in terms of degradation, we also studied the effect of peroxyl and superoxide radicals and found two pathways that had much lower activation barriers than the O_2_ adducts formed by Jovanovic- and Matsuda-type radicals. One of them, starting from the C4 adduct of KE-OOH^•^ (**30**) which is the most stable peroxyl adduct formed an epoxide species (**31**) after the peroxo bond cleavage (**TS**_**30–31**_). The generated free ^•^OH could coordinate to the C4 atom resulting in a very stable radical form (**32**) which gave the minor vanillin and 6-(4′-hydroxy-3′-methoxyphenyl)-2,4-dioxo-5-hexenal products (**33**) after a hydrogen atom loss in a barrierless step (see Scheme 8).

**Scheme 9:**
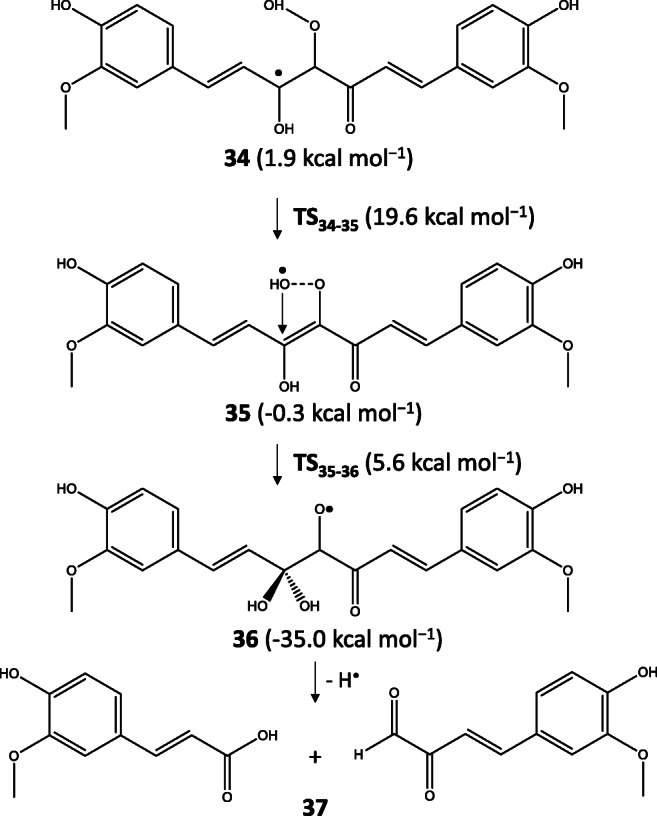
Proposed mechanism starting from the C1 adduct of the curcumin + peroxyl radical system resulting in ferulic acid and a feruloyl methane derivative

The other favored degradation pathway was starting from the C1 adduct of KE-OOH^•^ (**34**) which had 1.9 kcal mol^−1^ higher energy than the C4 adduct. From this, a peroxo bond cleavage occurred (**TS**_**34–35**_) which had a lower barrier by 1.8 kcal mol^−1^ than that of **TS**_**30**-**31**_. The free ^•^OH in **35** could coordinate to the C2 atom (**TS**_**35–36**_) resulting in a very stable radical species (**36**) from which after a hydrogen loss the major degradation product ferulic acid and the aldehyde derivative of feruloylmethane (**37**) formed (see Scheme 9).

As the relative energy of the degradation products showed, ferulic acid + feruloyl methane derivative was more stable by 5.3 kcal mol^−1^ than *o*-vanillin + *trans*-6-(4′-hydroxy-3′-methoxyphenyl)-2,4-dioxo-5-hexenal which means a thermodynamic preference of the major degradation products identified experimentally [34, 35]. The minor degradation products had relative energy of 22.9 kcal mol^−1^ while the major product had 17.6 kcal mol^−1^ referred to as the most stable product of the autoxidation process. These energy values were between the energies of spiroepoxide and vinylethercyclopentadione intermediates.

In the case of superoxide, the C1 adduct was more stable than the C4 adduct by 3.3 kcal mol^−1^ while the peroxo bond cleavage was also preferred for the C1 adduct by 4.9 kcal mol^−1^. All these results predict that peroxyl and superoxide radicals are responsible for the degradation instead of oxygen molecule.

## Conclusion

**Scheme 10:**
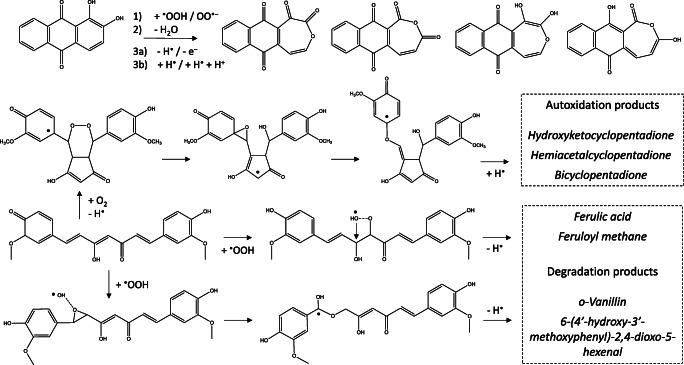
Summary of the most important species found by DFT calculations

In this work, we studied the antioxidant ability of two well-known natural compounds, alizarin and curcumin. Reactions with small harmful radicals such as hydroxyl, peroxyl, and superoxide radicals were investigated in detail by solvent models. We made a detailed mechanistic study, and found that hydroxyl radical can coordinate to the alizarin ring resulting in very stable adducts due to the possible orientation of the radical indicating further decomposition process. Peroxyl radical did not form stable adducts, but kinetically more preferred than hydrogen peroxide formation despite the latter one is thermodynamically favored. From the peroxyl adducts after the peroxo bond cleavage, different oxo heptacyclo species could form (see Scheme 10). These oxidation species are ether derivatives of the naturally occurring tropolones which have previously never been proposed. We found that all species are extremely favored thermodynamically; however, the formation of the more stable 1,3-dioxo heptacyclo species was kinetically preferred by superoxide radical. In biological systems, these products can be formed but further decompositions can be also proposed.

The autoxidation process of curcumin in which oxygen interacts with the radical forms of curcumin was studied in detail. It was found that the diketo isomer is responsible for the first steps due to the flexibility it compared to the rigid keto–enol isomer, which is in good agreement with the experimental results. We studied the possible pathways of the autoxidation through cyclic radical forms and found that the key intermediate is the epoxide form (see Scheme 10) from which all cyclopentadione could be formed in contrast to the experimentally proposed mechanism where only the bicyclopentadione pathway was assumed from the epoxide intermediate. However, the formation of hydroxyketocyclopentadione and hemiacetalcyclopentadione products was proposed before by ionic rearrangement; we showed that the mechanism including only radical species was much more favored and could explain some important questions. On the one hand, experimentally bicyclopentadione was found as a major product but our calculations showed that it had significantly higher energy compared to hydroxyketocyclopentadione and hemiacetalcyclopentadione which were in equilibrium experimentally and also by our calculations. This contradiction could be explained by the low activation barrier of the CC-cleavage of the epoxide unit resulted in the very stable vinylethercyclopentadione radical form (see Scheme 10). On the other hand, we concluded that the experimental appearance of the very unfavored spiroepoxide and vinylethercyclopentadione intermediates was based on the long-term rearrangements of hydrogen atoms, i.e., outer sphere hydrogen transfers.

The minor and major degradation products of curcumin could be found by some rearrangements starting from adducts formed by peroxyl and superoxide radicals. The activation barriers were much lower than those derived from the reaction between the open-chain radical form of curcumin and the oxygen molecule, which was experimentally proposed.

Comparing alizarin to curcumin, it can be concluded that the rigid alizarin can also transform into oxidized species by peroxyl or superoxide radicals. Activation barriers of the superoxide–alizarin pathways are similar to the barriers of curcumin’s autoxidation and degradation processes that propose a possible oxidation reaction in the case of alizarin as well.

## Supplementary information

ESM 1 (DOCX 1020 kb)
