# Supplementary material for: Radical scavenger competition of alizarin and curcumin: a mechanistic DFT study on antioxidant activity
Source: J Mol Model. 2021 May 13;27(6):166. doi: 10.1007/s00894-021-04778-1 (PMC8119285; doi:10.1007/s00894-021-04778-1)
Supplement: Supplementary file 1 — (DOCX 1020 kb) [file 894_2021_4778_MOESM1_ESM.docx]

**Supplementary Material**

**Radical scavenger competition of alizarin and curcumin: a mechanistic DFT study on antioxidant activity**

Malek Sadatsharifi^a^, Mihály Purgel^b,*^

^a^ University of Debrecen, Egyetem tér 1, H-4032 Debrecen, Hungary

^b^ Department of Physical Chemistry, University of Debrecen, Egyetem tér 1, H-4032 Debrecen, Hungary

*Corresponding author: Mihály Purgel

E-mail address: [purgel.mihaly@science.unideb.hu](mailto:purgel.mihaly@science.unideb.hu)

**Figure S1** Optimized structures of the proposed products of the alizarin + peroxyl/superoxide radical systems in different orientations.

**Figure S2** Optimized structures of curcumin radicals in different orientations. Blue shaded ovals represent the unfavored structure of quinone ring.

**Table S1** Relative Gibbs free energies in kcal mol^–1^ by *in vacuo*, IEF-PCM and SMD models at M06/TZVP level for AR-OH^•^ and AR-OOH^•^ adducts referred to the alizarin + outer sphere hydroxyl/peroxyl radical. Very unfavored species (over 10 kcal mol^–1^ *in vacuo* referred to the most stable adduct) were not re-optimized by implicit solvent models.

| adduct | *in vacuo* | IEF-PCM | SMD |
| --- | --- | --- | --- |
| AR-OH^C3•^ | -11.4 | -19.2 | -19.9 |
| AR-OH^C4•^ | -13.5 | -22.6 | -24.5 |
| AR-OH^C5•^ | -4.9 | -16.3 | -17.8 |
| AR-OH^C6•^ | -12.5 | -21.0 | -21.3 |
| AR-OH^C7•^ | -6.2 | -14.2 | -14.9 |
| AR-OH^C10•^ | -9.1 | -16.5 | -16.5 |
| AR-OH^C11•^ | -6.7 | -16.3 | -17.6 |
| AR-OH^C12•^ | -5.7 | -15.6 | -17.1 |
| AR-OH^C13•^ | -9.4 | -16.9 | -17.3 |
| AR-OOH^C3•^ | 12.4 | 13.1 | 9.7 |
| AR-OOH^C4•^ | 9.8 | 10.6 | 6.7 |
| AR-OOH^C5•^ | 16.6 | 14.8 | 11.5 |
| AR-OOH^C6•^ | 10.2 | 10.2 | 7.3 |
| AR-OOH^C7•^ | 15.8 | 16.3 | 13.5 |
| AR-OOH^C10•^ | 13.6 | 14.0 | 10.8 |
| AR-OOH^C11•^ | 14.6 | 14.5 | 10.7 |
| AR-OOH^C12•^ | 15.4 | 15.2 | 11.3 |
| AR-OOH^C13•^ | 14.1 | 13.5 | 11.0 |

**Table S2** Relative Gibbs free energies in kcal mol^–1^ by *in vacuo*, IEF-PCM, and SMD models at M06/TZVP level for AR-superoxide (AR-OO^•–^) adducts referred to the AR + outer sphere superoxide. Species over 10 kcal mol^–1^ *in vacuo* were not re-optimized by implicit solvent models.

| starting adduct | *in vacuo* | IEF-PCM | SMD |
| --- | --- | --- | --- |
| AR-OO^C1•–^ | 0.0^*^ | 1.3^*^ | 0.0^*^ |
| AR-OO^C2•–^ | 0.0^*^ | 14.1^⁑^ | 32.1 |
| AR-OO^C3•–^ | 1.3^*^ | 1.6^*^ | 24.9 |
| AR-OO^C4•–^ | 2.9^**^ | 20.8 | 17.6 |
| AR-OO^C5•–^ | 5.5^⁑^ | 6.2^⁑^ | 5.6^⁑^ |
| AR-OO^C7•–^ | 0.4^*^ | 34.3 | 27.8 |
| AR-OO^C8•–^ | 1.0^*^ | 3.0^*^ | 1.2^*^ |
| AR-OO^C9•–^ | 0.4^*^ | 38.1 | 31.7 |
| AR-OO^C12•–^ | 1.1^*^ | 0.0^*^ | 27.2 |
| AR-OO^C14•–^ | 0.0^*^ | 2.6^*^ | 32.5 |
| generated system | *in vacuo* | IEF-PCM | SMD |
| AR^•–^ + O_2_ | 3.5 | 6.1 | 6.5 |
| AR^-H^-OOH^C4•–^ | 4.3^⁑^ | 3.4^⁑^ | -0.3^⁑^ |
| AR^-H^O^C4^-OH^C6•–^ | -9.2 | -2.2 | -15.1 |

^*^dissociated superoxide radical from the adduct

^**^dissociated superoxide radical from the adduct and bonded by hydrogen bond

^⁑^coordinated superoxide radical and intramolecular hydrogen transfer

**Table S3** Relative Gibbs free energies in kcal mol^–1^ by in vacuo, IEF-PCM and SMD models at M06/TZVP level for DK/KE-OH• and DK/KE-OOH• adducts of curcumin referred to the curcumin + outer sphere hydroxyl/peroxyl radical. Very unfavored species (over 10 kcal mol^–1^ *in vacuo* referred to the most stable adduct) were not re-optimized by implicit solvent models.

| adduct | *in vacuo* | IEF-PCM | SMD |
| --- | --- | --- | --- |
| DK-OH^C3•^ | -22.1 | -27.7 | -22.4 |
| DK-OH^C4•^ | -21.9 | -26.6 | -19.6 |
| KE-OH^C1•^ | -27.5 | -32.0 | -24.8 |
| KE-OH^C3•^ | -23.9 | -28.8 | -22.4 |
| KE-OH^C4•^ | -26.9 | -30.1 | -23.3 |
| KE-OH^C6•^ | -19.7 | -22 | -15.7 |
| KE-OH^C8•^ | -22.3 | -26.6 | -21.0 |
| DK-OOH^C3•^ | 0.0* | 0.0* | 0.0* |
| DK-OOH^C4•^ | 6.6 | 5.0 | 5.5 |
| KE-OOH^C1•^ | -1.0 | -1.2 | 2.4 |
| KE-OOH^C3•^ | 2.2 | 0.5 | 1.3 |
| KE-OOH^C4•^ | -0.5 | -0.9 | 1.2 |
| KE-OOH^C6•^ | 7.2 | 5.6 | 5.8 |
| KE-OOH^C8•^ | 4.8 | 4.0 | 4.1 |

^*^dissociated OOH-radical from the adduct, therefore, it is the reference

**Table S4** Relative Gibbs free energies in kcal mol^–1^ by *in vacuo*, IEF-PCM, and SMD models at M06/TZVP level for curcumin-superoxide (DK/KE-OO^•–^) adducts. Note that species over 12 kcal mol^–1^ by *in vacuo* were not reoptimized by implicit solvent models.

| starting adduct | *in vacuo* | IEF-PCM | SMD |
| --- | --- | --- | --- |
| DK-OO^C8•–^ | 3.3^⁂^ | 19.5^⁑^ | 17.5 |
| KE-OO^C1•–^ | 11.3^⁑^ | 7.1^⁑^ | 16.9 |
| KE-OO^C8•–^ | 0.0^⁂^ | 0.0^⁂^ | 17.6 |
| KE-OO^C9•–^ | 9.0^⁑^ | 11.0^⁑^ | 11.5 ^⁑^ |
| generated system | *in vacuo* | *IEF-PCM* | *SMD* |
| KE^•–^ + O_2_ | 19.5 | 19.8 | 19.2 |
| KE‧‧‧O_2_^•–^ | – | – | 0.0 |

^⁑^coordinated superoxide radical and intramolecular hydrogen transfer

^⁂^dissociated superoxide radical from adduct and intermolecular proton transfer

**One unit model vs. separated molecular model**

In this work, we use the „one unit model” (OUM), in which molecules interact with each other in contrast to the separate molecular model (*sep*). OUM means that we count with a reference system where reactant molecules are close enough to each other to interact whereas in *sep* we would count with “pure material”. OUM reference structures were AR/CM···OH^•^, AR/CM···OOH^•^, AR···O_2_^•–^ and [AR + O_2_^•–^] for the reactions (1), (3), (5) and (7), respectively. The latter means that the superoxide radical was “over” the aromatic ring of AR, having a similar orientation like between AR^•–^and O_2_. ΔG of OUM and *sep* in the case of (1) and (3) showed good agreement, however; for (5) and (7) the exergonic-endergonic relation was turned, see Table 1. It is known that due to the difference in translational entropy [54], the sum of separated molecules’ energy is lower than interacted molecules but this phenomenon is true for both reactant and product sides. Stationary points of the mechanisms referred to the separated molecules (OUM*) have an energy-shift ~5 kcal mol^–1^, see Table S5–S7. In solution, it is a fact that there are molecules separated by the bulk and also interacted species which went through diffusion strongly depending on concentration. Obviously, it is hard (or impossible) to define the ratio of OUM and *sep* in a real system, not to mention the real explicit solvent effect.

**Table S5** Reaction Gibbs free energies of alizarin-small radical systems. *sep* and OUM are related to the molecular model defined in the article. Energies of *sep* and OUM* are referred to as separated AR + ^•^OH/^•^OOH/O_2_^•–^ systems.

|  | *sep* | OUM | OUM* |
| --- | --- | --- | --- |
| AR/CM + OH^•^ → AR/CM^•^ + H_2_O | -31.8 | -32.2 | -26.8 |
| AR/CM + ^•^OOH → AR/CM^•^ + H_2_O_2_ | 0.8 | 1.4 | 5.9 |
| AR + O_2_^•–^ → AR^•–^ + ^•^OOH | -0.5 | -0.8 | 6.1 |
| AR + O_2_^•–^ → AR^•–^ + O_2_ | 3.0 | 1.4 | -1.0 |

**Table S6** Relative free energies of alizarin-small radical systems at M06/TZVP level. Position (carbon atom) of the formation of most stable intermediate adducts are assigned as a superscript. *sep* and OUM are related to the molecular model defined in the article. Energies of *sep* and OUM* are referred to separated AR + ^•^OH/^•^OOH/O_2_^•–^ showing higher activation barriers than OUM.

AR + OH^•^ → AR^•^ + H_2_O (1)

AR + ^•^OH → AR-OH^•^ (1a)

AR + ^•^OOH → AR^•^ + H_2_O_2_ (2)

AR + OOH^•^ → AR-OOH^•^ (2a)

AR + ^•^OOH → ARO-OH^•^ (2’)

AR + ^•^OOH → ARO + ^•^OH (2a’)

AR + O_2_^•–^ → AR^•–^ + O_2_ (3)

|  | ΔG^‡o.v.^ | | | ΔG^o.v.^ | | |
| --- | --- | --- | --- | --- | --- | --- |
|  | *in vacuo* | IEF-PCM | SMD | *in vacuo* | IEF-PCM | SMD |
| (1) *sep* | – | – | – | -30.9 | -35.0 | -36.0 |
| (1) OUM | 6.3 | – | 0.4 | -23.1 | -36.8 | -36.8 |
| (1) OUM* | 2.9 | 5.7 | 7.2 | -26.4 | -30.4 | -30.0 |
| (1a)^C4^ OUM | 8.1 | -1.1 | -2.7 | -13.5 | -22.6 | -24.5 |
| (1a)^C4^ OUM* | 4.8 | 5.4 | 4.1 | -16.8 | -16.2 | -17.7 |
| (2) *sep* | – | – | – | 1.4 | -2.1 | -3.2 |
| (2) OUM | 13.7 | 13.0 | 11.3 | 3.9 | -0.9 | -3.4 |
| (2) OUM* | 15.8 | 16.9 | 18.1 | 6.1 | 3.0 | 3.4 |
| (2a)^C4^ OUM | – | – | – | 9.8 | 10.2 | 6.7 |
| (2a)^C6^ OUM | – | – | – | 10.1 | 9.8 | 7.4 |
| (2a)^C4^ OUM* | – | – | – | 11.9 | 14.1 | 13.5 |
| (2a)^C6^ OUM* | – | – | – | 12.3 | 13.6 | 14.1 |
| (2’)^C6^ OUM | 36.4 | 34.0 | 28.4 | -25.1 | -24.6 | -26.4 |
| (2a’)^C4^ OUM | 26.4 | 22.7 | 18.3 | 6.2 | – | – |
| (2’)^C6^ OUM* | 38.6 | 37.9 | 35.2 | -23.0 | -20.7 | -19.7 |
| (2a’)^C4^ OUM* | 28.5 | 26.6 | 25.0 | 8.3 | – | – |
| (3) *sep* | – | – | – | -40.5 | -6.4 | 1.5 |
| (3) OUM | – | – | – | 3.6 | 6.1 | 6.5 |
| (3) OUM* | – | – | – | -33.6 | 1.2 | 9.5 |

**Table S7** Relative free energies of curcumin-small radical systems at M06/TZVP level. Position (carbon atom) of the formation of most stable intermediate adducts are assigned as a superscript. *sep* and OUM are related to the molecular model defined in the article. Energies of *sep* and OUM* are referred to separated KE + ^•^OH/^•^OOH/O_2_^•–^ showing higher activation barriers than OUM.

CM + ^•^OH → CM^•^ + H_2_O (4)

CM + ^•^OH → CM-OH^•^ (4a)

CM + ^•^OOH → CM^•^ + H_2_O_2_ (5)

CM + ^•^OOH → CM-OOH^•^ (5a)

CM + O_2_^•–^ → CM^•–^ + O_2_ (6)

|  | ΔG^‡o.v.^ | | | ΔG^o.v.^ | | |
| --- | --- | --- | --- | --- | --- | --- |
|  | *in vacuo* | IEF-PCM | SMD | *in vacuo* | IEF-PCM | SMD |
| (4)^KE^ *sep* | – | – | – | -37.8 | -41.0 | -42.0 |
| (4)^KE^ OUM | – | – | – | – | -43.8 | – |
| (4)^KE^ OUM* | – | – | – | -37.0 | -36.5 | -36.1 |
| (4a)^KE-C1^ OUM | – | ^n.c.^ | – | – | -32.0 | – |
| (4a)^KE-C1^ OUM* | ^n.c.^ | ^n.c.^ | ^n.c.^ | -27.5 | -24.7 | -24.8 |
| (5)^KE^ *sep* | – | – | – | -5.6 | -8.0 | -9.3 |
| (5)^KE^ OUM | 10.4 | 7.7 | 9.2 | -4.2 | -10.8 | -8.9 |
| (5)^KE^ OUM* | 12.4 | 14.4 | 14.6 | -2.3 | -4.2 | -3.4 |
| (5a)^CM-CX^ OUM | ^n.c.^ | ^n.c.^ | ^n.c.^ | -1.0^KE-C4^ | -1.2^KE-C4^ | 0.5^DK-C1^ |
| (5a)^CM-CX^ OUM* | ^n.c.^ | ^n.c.^ | ^n.c.^ | 0.9^KE-C4^ | 5.4^KE-C4^ | 6.0^DK-C1^ |
| (6)^KE^ *sep* | – | – | – | -29.7^#^ | 6.1 | 13.5 |
| (6)^KE^ OUM | – | – | – | 19.6 | 19.9 | 19.2 |
| (6)^KE^ OUM* | – | – | – | -21.5^#^ | 15.0 | 21.6 |

^#^refers to the huge effect of absence of solvation

^CM-CX^refers to different coordination modes of curcumin

**NUMBER OF ATOMS, ENERGIES AND COORDINATES OF THE MOST RELEVANT STRUCTURES OPTIMIZED BY IEF-PCM**

Number of atoms: 29

Symbol (number/name) of the species: 1

Energies in atomic unit (Hartree):

Electronic energy:

E(SCF) = -990.122392045,

Zero point corrected energy:

E(ZPE) = -989.915754,

Gibbs free energy:

E(G) = -989.959478 a.u.

Cartesian coordinates:

C 4.73389400 0.08507200 0.23856700

C 3.71448900 1.02396900 0.20138800

C 2.39213700 0.61374600 0.06142300

C 2.10172400 -0.75379300 -0.03718000

C 3.13042600 -1.69199000 0.00039700

C 4.44329400 -1.27269200 0.13816800

C 1.31414800 1.63861200 0.01267000

C 0.70774200 -1.21421200 -0.18429400

C -0.35418300 -0.21016200 -0.17045400

C -0.05869000 1.17802200 -0.11416900

C -1.11436300 2.13688500 -0.23278700

H -0.84638900 3.18425900 -0.27430200

C -2.40149300 1.76678800 -0.31651300

C -2.83038900 0.34079200 -0.19470700

C -1.66650600 -0.63096700 -0.27932200

H 5.76033100 0.41154200 0.34711500

H 3.93547500 2.08032400 0.27897500

H 2.89274600 -2.74480300 -0.07799000

H 5.24298200 -2.00149800 0.16876400

H -3.20583100 2.48298100 -0.42520300

O 0.44220000 -2.41630400 -0.31006000

O 1.58503200 2.83946400 0.07447500

O -2.01326300 -1.88173100 -0.43638400

H -1.16783400 -2.42376900 -0.44406400

O -3.79783200 0.05602600 -1.15513300

H -4.04301000 -0.88022600 -1.08559100

O -3.39542900 0.28258000 1.12906500

O -4.05007700 -0.96703700 1.31497100

H -3.40690100 -1.47321800 1.83769300

29

2, E(SCF) = -990.199218251, E(ZPE) = -989.995623, E(G) = -990.042959 a.u.

C 4.62424400 -0.40663000 0.44199000

C 3.73555300 0.65552600 0.48415500

C 2.39319700 0.45248000 0.17670600

C 1.95147600 -0.82616000 -0.17039500

C 2.85012600 -1.89136300 -0.21820200

C 4.18290400 -1.68038200 0.08962100

C 1.45504000 1.59917200 0.20129900

C 0.53675100 -1.06335300 -0.48974000

C -0.43955000 0.03728500 -0.31448700

C 0.04276100 1.36409300 -0.08309700

C -0.74004300 2.54359700 -0.23026500

H -0.18126300 3.46717300 -0.31053600

C -2.06519900 2.71312800 -0.30343100

C -2.98037700 0.47147400 0.03076900

C -1.77188100 -0.32250100 -0.41009100

H 5.66712900 -0.24404800 0.68285900

H 4.07354600 1.64711900 0.75372200

H 2.49562300 -2.87621800 -0.49158800

H 4.88102700 -2.50678600 0.05759500

H -2.50734500 3.68385000 -0.47123400

O 0.17305700 -2.18146400 -0.87330100

O 1.87245300 2.73242500 0.45234200

O -2.16349600 -1.52354200 -0.75678400

H -1.32096400 -2.06412100 -0.93754000

O -3.94072200 -0.11117000 0.45301100

H -3.89376900 -2.02365900 0.90225300

O -3.06593200 1.80193300 -0.09049500

O -3.94744300 -2.91116800 1.28813900

H -3.28730600 -3.41761400 0.80218000

29

3, E(SCF) = -990.205101914, E(ZPE) = -989.999567, E(G) = -990.045714 a.u.

C 4.46911100 0.16426200 0.39510500

C 3.45676600 1.10373900 0.40145300

C 2.14747500 0.72571700 0.09740500

C 1.86991300 -0.61377700 -0.21508000

C 2.90595600 -1.55925400 -0.22791900

C 4.19222500 -1.16986200 0.08091500

C 1.07567500 1.73234100 0.09617600

C 0.51980300 -0.99755200 -0.54202600

C -0.54328500 -0.08080300 -0.44198000

C -0.28780300 1.28412500 -0.16978700

C -1.26130300 2.33233200 -0.26695800

H -0.85268100 3.32537500 -0.40496200

C -2.59492000 2.30732400 -0.21057300

C -3.09763800 -0.03715300 0.14713500

C -1.88606000 -0.61609300 -0.61165900

H 5.48236300 0.46129900 0.63403400

H 3.66445600 2.13853900 0.63952500

H 2.68804400 -2.58953400 -0.47356100

H 4.99121600 -1.90012700 0.07768200

H -3.18897500 3.19960800 -0.34004900

O 0.35922300 -2.25344500 -0.91301200

O 1.32423500 2.92531800 0.32671100

O -2.12818700 -1.65905400 -1.19667200

H -0.57075600 -2.40620200 -1.19109700

O -3.81607900 -0.79654000 0.73340200

H -2.49315800 -2.25177900 1.60848100

O -3.42138200 1.25904500 0.10624900

O -1.59903100 -2.53356300 1.84450200

H -1.26621900 -2.96335200 1.04725800

27

4, E(SCF) = -914.358871024, E(ZPE) = -914.166122, E(G) = -914.208345 a.u.

C 4.44607500 0.24023200 0.16698600

C 3.38412300 1.12601200 0.04746600

C 2.08426400 0.63927300 -0.01266500

C 1.85081500 -0.73548700 0.04010700

C 2.91654200 -1.62023600 0.15978500

C 4.21300700 -1.12992100 0.22092200

C 0.94720500 1.58129100 -0.15775700

C 0.46699500 -1.26009700 -0.00107900

C -0.67428400 -0.30221800 -0.14181300

C -0.43874800 1.04002100 -0.19889700

C -1.48017000 2.03160900 -0.42023300

H -1.23364000 2.89971500 -1.01816000

C -2.71153300 1.93465700 0.06094300

C -3.09562700 -0.29361900 0.30412500

C -2.00086600 -0.89082900 -0.20151200

H 5.45893500 0.61870500 0.21721000

H 3.55702700 2.19325600 0.00346400

H 2.72516200 -2.68429900 0.20341700

H 5.04387400 -1.81742900 0.31364500

H -3.51454400 2.63427400 -0.12963100

O 0.25329600 -2.45866800 0.09166400

O 1.13660900 2.78148900 -0.25433600

O -2.22235700 -2.10971700 -0.82906600

O -4.32432500 -0.81943000 0.28269600

O -3.05360000 0.90456500 0.95016100

H -1.51072500 -2.71143500 -0.55384900

H -4.28987800 -1.68284000 -0.16331900

28

5, E(SCF) = -989.681463945, E(ZPE) = -989.487049, E(G) = -989.530947 a.u.

C 4.66839000 0.15952800 0.28673700

C 3.62831400 1.06766900 0.21489500

C 2.31484600 0.62376600 0.04138800

C 2.05472500 -0.74881200 -0.05865100

C 3.11272500 -1.65701700 0.01939900

C 4.40975800 -1.20822800 0.18982000

C 1.21556600 1.60867200 -0.03903700

C 0.67148100 -1.26085900 -0.25150200

C -0.39060600 -0.27878600 -0.30015600

C -0.12262000 1.10238900 -0.20508400

C -1.18821300 2.07566600 -0.34961600

H -0.89863300 3.11264800 -0.45795800

C -2.47821300 1.73053600 -0.36447700

C -2.86843100 0.31114200 -0.13173300

C -1.75919100 -0.70173600 -0.50184200

H 5.68398600 0.51096900 0.41902500

H 3.81907000 2.13014600 0.29088800

H 2.90435500 -2.71599200 -0.05706000

H 5.22471500 -1.91871800 0.24840700

H -3.28004900 2.44810100 -0.48395900

O 0.48234300 -2.48334900 -0.34217700

O 1.45756200 2.83195000 0.03695800

O -2.13344600 -1.79843100 -0.90673400

O -4.04839200 -0.00387700 -0.79219100

H -4.03397600 -0.96226300 -0.95595600

O -3.06239600 0.25146600 1.29979900

O -3.48954100 -1.05933000 1.66341600

H -2.67969000 -1.45590500 2.02321700

28

6, E(SCF) = -989.665611519, E(ZPE) = -989.472594, E(G) = -989.517599 a.u.

C -4.76139000 -0.02121800 -0.16736800

C -3.76703500 0.94550900 -0.14108300

C -2.43369700 0.56215600 -0.04688500

C -2.09706300 -0.79277500 0.01962500

C -3.09695300 -1.75686500 -0.01673400

C -4.42663600 -1.36979600 -0.10607800

C -1.37518100 1.59780800 -0.02282300

C -0.67370000 -1.21868900 0.10799600

C 0.35729700 -0.16053200 0.22398100

C 0.05381800 1.15219100 0.11331100

C 1.07329500 2.19578100 0.13656700

H 0.73810100 3.22372100 0.13418700

C 2.36917400 1.88625400 0.11315200

C 2.84641700 0.45911500 0.02821100

C 1.77070700 -0.52215100 0.53303200

H -5.79962300 0.27660300 -0.23780400

H -4.01670600 1.99731300 -0.19355800

H -2.82936500 -2.80444400 0.02948900

H -5.20536000 -2.12149400 -0.12931900

H 3.14210000 2.64557700 0.07445500

O -0.36838100 -2.39555400 0.05383400

O -1.63150200 2.78198200 -0.09922600

O 2.06252800 -1.53328900 1.13058400

O 4.02506700 0.32532100 0.78336900

H 4.40998100 -0.50157400 0.42095500

O 3.04997000 0.16223000 -1.30684900

O 4.24889600 -1.63007500 -1.03807800

H 3.43392700 -2.14133600 -0.93690900

28

7, E(SCF) = -989.787529866, E(ZPE) = -989.595020, E(G) = -989.641756 a.u.

C 4.81256600 0.15674900 0.12516300

C 3.76298100 1.05137900 0.03485200

C 2.44519700 0.58862800 -0.01261200

C 2.19161600 -0.78674000 0.03181400

C 3.25907300 -1.68265500 0.12410100

C 4.55931400 -1.21525600 0.16965300

C 1.33730100 1.56079200 -0.11478600

C 0.80693500 -1.31254900 -0.01678300

C -0.25966400 -0.33907400 -0.05843700

C -0.02724100 1.04390400 -0.13062100

C -1.03807000 2.03366600 -0.38289700

H -0.63392400 2.97726100 -0.73220200

C -2.38558400 1.99351600 -0.27753500

C -3.17295200 0.96150100 0.35495500

C -1.61472100 -0.86730900 -0.06470000

H 5.83156400 0.52061400 0.16215200

H 3.94879700 2.11668600 0.00129400

H 3.05462800 -2.74438000 0.15956300

H 5.38240000 -1.91502400 0.24204300

H -2.96212100 2.85987800 -0.57217300

O 0.60949000 -2.53869700 0.01443000

O 1.57853300 2.77617500 -0.18006400

O -2.00520000 -1.87019200 -0.60052100

O -4.34571100 1.06774300 0.64062500

H -4.04849700 -1.94038200 -0.41423500

O -2.54576400 -0.19805600 0.74196600

O -4.99395900 -1.74912700 -0.32531100

H -5.01247900 -0.89672000 0.13156600

27

9, E(SCF) = -914.397418286, E(ZPE) = -914.205034, E(G) = -914.246667 a.u.

C 4.41852600 0.51871600 -0.10017300

C 3.29903900 1.30981500 0.09149200

C 2.02900200 0.73821400 0.09141300

C 1.88759400 -0.64319500 -0.09538800

C 3.02202600 -1.43659900 -0.27981800

C 4.27775500 -0.85463000 -0.28799200

C 0.83878900 1.60292000 0.27178000

C 0.56105100 -1.25178800 -0.04037100

C -0.57826700 -0.48501200 0.07922800

C -0.48256000 0.97818600 0.07583100

C -1.47747100 1.82824700 -0.29273000

H -1.19473300 2.87023700 -0.39432600

C -2.83125000 1.52329900 -0.62395900

C -3.47554500 0.44635300 -0.14920700

C -1.81535000 -1.19375600 0.29862000

H 5.40349900 0.96767300 -0.10737400

H 3.39637400 2.37756400 0.23786800

H 2.91435900 -2.50363900 -0.41707400

H 5.15379700 -1.47259000 -0.43838900

H -3.40324800 2.21990400 -1.22043500

O 0.56613200 -2.57755900 -0.07620500

O 0.96917300 2.79867100 0.51719900

O -1.96761100 -2.39613400 0.25978300

O -4.75776900 0.20095500 -0.40660300

O -2.92422500 -0.45362300 0.71684400

H -5.03635100 -0.61652000 0.03545500

H -0.35933100 -2.90572200 -0.01623700

46

10, E(SCF) = -1262.88990547, E(ZPE) = -1262.531825, E(G) = -1262.590001 a.u.

C 0.06838500 0.07731800 0.04196400

C -1.17123300 -0.65680700 0.08541000

C 1.27432000 -0.56582500 0.01812700

C 2.52311700 0.16889200 -0.03096200

H 2.42645000 1.24630900 -0.07775900

C -2.41146700 0.12387200 0.11705600

H -2.30491300 1.20090900 0.16574900

C 3.72124000 -0.44997200 -0.01032700

H 3.74170500 -1.53232600 0.06439100

C -3.61013400 -0.47510500 0.07005100

H -3.63112100 -1.55931000 -0.00580900

O -1.18061000 -1.91064600 0.09242800

O 1.36608800 -1.89784200 0.03466800

C 5.00772800 0.19734600 -0.06916700

C 6.15478700 -0.60280100 0.06579000

C 5.14574700 1.60608800 -0.27109200

C 7.41029900 -0.04070400 0.01787000

H 6.02848900 -1.66671700 0.21701000

C 6.37013500 2.18277700 -0.32626300

H 4.26125400 2.21900400 -0.38542300

C 7.58008900 1.40344000 -0.18906900

H 6.49126300 3.24735900 -0.48386900

C -4.91420200 0.17740600 0.09876900

C -6.05378600 -0.61841700 -0.10231800

C -5.07126300 1.54615100 0.32371600

C -7.31359900 -0.05105200 -0.08846500

H -5.93040600 -1.68004700 -0.27372300

C -6.33501000 2.11513000 0.34057200

H -4.20826900 2.17711400 0.49302900

C -7.45430600 1.32533100 0.13916100

H -6.47089700 3.17427300 0.51986900

O -8.48884900 -0.71616500 -0.27690400

O -8.69333500 1.89161600 0.16428900

H -9.35582900 1.20344500 -0.00085900

O 8.71431600 1.91213400 -0.24022200

O 8.55889500 -0.70556200 0.14345600

C 8.49960900 -2.11893600 0.34924700

H 9.53120800 -2.45132300 0.41366000

H 7.97506200 -2.34444100 1.27893600

H 8.00298400 -2.60517900 -0.49139400

C -8.41857700 -2.12607400 -0.46640100

H -7.85289300 -2.36691400 -1.36849600

H -7.96178600 -2.60892000 0.39970300

H -9.44468000 -2.46599300 -0.57614000

H 0.04894500 1.15709600 0.02902500

H 0.43011800 -2.24096800 0.05839000

46

11, E(SCF) = -1262.88905813, E(ZPE) = -1262.530500, E(G) = -1262.586352 a.u.

C 4.94383200 -0.00263000 0.00030100

C 4.10042700 -1.22882100 0.32117000

C 4.09404300 1.20582600 -0.35099900

C 2.87337300 1.38325700 0.46286800

C 2.88325000 -1.38414000 -0.49000100

H 2.87937800 -0.90656600 -1.46411700

C 1.74086600 1.82640200 -0.10999800

H 1.78313100 2.15810600 -1.14417400

C 1.75533100 -1.84111300 0.07080700

H 1.80788100 -2.21745700 1.09003400

O 4.39763200 -1.97229300 1.24076100

O 4.37661000 1.93482500 -1.28426700

C 0.42548500 1.73828100 0.48128300

C -0.68638200 1.88754800 -0.36017400

C 0.23611200 1.41299800 1.86067400

C -1.96051700 1.67178500 0.12085700

H -0.52050900 2.13175700 -1.40180600

C -1.00665700 1.22060400 2.36491800

H 1.09790200 1.32539900 2.50988500

C -2.18669300 1.33503700 1.53479600

H -1.16760700 0.98205700 3.40928600

C 0.41919100 -1.73253000 -0.50304800

C -0.67955600 -1.86412900 0.36201100

C 0.19693000 -1.40176200 -1.84191900

C -1.96262600 -1.64238900 -0.10096100

H -0.50638800 -2.11907700 1.40022500

C -1.09151800 -1.20108800 -2.31271200

H 1.02995800 -1.30592200 -2.52679700

C -2.17144400 -1.31130700 -1.45095300

H -1.27743700 -0.95300200 -3.35043900

O -3.09811200 -1.70809800 0.64457900

O -3.42934500 -1.10139100 -1.91426200

H -4.05706900 -1.17341500 -1.17808800

O -3.33606000 1.15893300 1.97220800

O -3.07760700 1.74239300 -0.59951900

C -2.96969400 2.09783400 -1.97883100

H -3.98253500 2.08064000 -2.36982300

H -2.54652000 3.09881100 -2.07772000

H -2.35141300 1.37537000 -2.51445800

C -2.96335100 -2.06939400 2.01490100

H -2.55163900 -3.07685000 2.10569400

H -2.32417000 -1.35988500 2.54489600

H -3.96535300 -2.04094900 2.43415300

H 5.56038600 0.22844900 0.87213500

H 5.59933000 -0.22234100 -0.84550400

H 2.88531700 0.95832400 1.46067000

46

12, E(SCF) = -1262.87762607, E(ZPE) = -1262.518448, E(G) = -1262.573595 a.u.

C 5.08607700 -0.21341800 -0.06721500

C 4.09816500 -1.29021900 -0.44180200

C 4.23612700 0.95684900 0.35695600

C 2.79153100 0.50818200 0.50663800

C 2.68925700 -0.71178100 -0.47996800

H 2.58521500 -0.29790200 -1.49153600

C 1.79507000 1.57187600 0.22550300

H 2.10123600 2.32406200 -0.49782400

C 1.61747000 -1.68264400 -0.15689300

O 4.37242600 -2.44332300 -0.67151900

O 4.62779100 2.08458500 0.53652700

C 0.52216400 1.60693500 0.67317200

C -0.40037000 2.57098700 0.09283100

C -0.01699900 0.61110800 1.58489100

C -1.72896200 2.44457700 0.26344400

H 0.01943500 3.34532900 -0.53647100

C -1.33971600 0.48756000 1.77457100

H 0.66550300 -0.05798200 2.09488900

C -2.29224100 1.34617100 1.08153500

H -1.75794700 -0.27106900 2.42522900

C 0.27801700 -1.58121800 -0.58771000

C -0.69970600 -2.41717900 0.02135000

C -0.16660200 -0.61828200 -1.52259800

C -2.03649300 -2.23926400 -0.24086300

H -0.37229500 -3.16038500 0.73692300

C -1.51422800 -0.45317500 -1.78695300

H 0.54593800 0.02906100 -2.01690800

C -2.45556300 -1.24451000 -1.14700700

H -1.85778000 0.30181500 -2.48406300

O -3.06010900 -2.94342700 0.32327200

O -3.78294500 -1.05178400 -1.39279800

H -4.29548900 -1.66741200 -0.84690700

O -3.50878700 1.18360600 1.17252000

O -2.68545200 3.23570200 -0.27576500

C -2.23654900 4.31023500 -1.09502400

H -3.13039800 4.83378600 -1.42255500

H -1.59860900 4.98792500 -0.52341200

H -1.69040300 3.92953200 -1.96101500

C -2.72193000 -3.89422600 1.32771400

H -2.08991900 -4.68333700 0.91564900

H -2.21084900 -3.40553400 2.15992400

H -3.66212700 -4.31789500 1.66992100

H 5.73053700 -0.54086000 0.75266600

H 5.72460600 0.04647600 -0.91476700

H 2.70658900 0.10965900 1.52415900

H 1.85624500 -2.43952800 0.58275600

46

13, E(SCF) = -1262.87692094, E(ZPE) = -1262.516767, E(G) = -1262.571467 a.u.

C 4.94391000 -0.33077700 -0.14618500

C 4.02761100 -1.29623900 -0.71144900

C 4.23151900 0.60113200 0.51969100

C 2.75394500 0.33275300 0.55021500

C 2.59938400 -0.77548000 -0.55014800

H 2.38766900 -0.26366200 -1.49432400

C 1.87773600 1.49979700 0.26160200

H 2.27486300 2.23716300 -0.43211200

C 1.56334900 -1.78713300 -0.23577300

O 4.30799700 -2.35086400 -1.26507200

O 4.65961100 1.67182800 1.18054800

C 0.58764000 1.61746300 0.64655400

C -0.23807900 2.64936900 0.03830900

C -0.06448400 0.64799000 1.51320600

C -1.57873200 2.62241200 0.15642000

H 0.26163900 3.39523400 -0.56688100

C -1.39884000 0.62707100 1.65423500

H 0.53780400 -0.08560200 2.03374300

C -2.25549500 1.57411200 0.95352200

H -1.89764800 -0.10758600 2.27491000

C 0.19825100 -1.65329400 -0.57848000

C -0.75443600 -2.49775400 0.05714500

C -0.28765600 -0.65929100 -1.45910100

C -2.10241200 -2.29992200 -0.12691700

H -0.39740800 -3.26841100 0.72795700

C -1.64545000 -0.47230100 -1.64253400

H 0.39999100 -0.00469200 -1.97797500

C -2.55954900 -1.27438900 -0.97791700

H -2.01693100 0.30574200 -2.29904200

O -3.10041900 -3.01766900 0.46550700

O -3.89697100 -1.06606700 -1.14927600

H -4.38464900 -1.69175700 -0.59249400

O -3.48368100 1.52195400 1.01954800

O -2.45059000 3.48500000 -0.41657900

C -1.89213700 4.51386300 -1.22654000

H -2.73171100 5.09431500 -1.59878800

H -1.23313500 5.15340300 -0.63519000

H -1.33792500 4.08479200 -2.06437000

C -2.71504500 -4.05830900 1.35736200

H -2.12369900 -4.81341000 0.83571000

H -2.14600600 -3.65386800 2.19699300

H -3.63890600 -4.50138900 1.71907000

H 2.52858000 -0.11186200 1.52231900

H 1.83887000 -2.57761100 0.45435000

H 6.02004700 -0.38347500 -0.22606800

H 5.62479200 1.75278600 1.12549900

46

14, E(SCF) = -1262.86085331, E(ZPE) = -1262.498320, E(G) = -1262.552854 a.u.

C 5.06238800 0.12148500 0.03247200

C 4.11138500 1.03210500 -0.77002000

C 4.15429700 -0.84852300 0.81410800

C 2.82696800 -0.71189000 0.12962700

C 2.77999700 0.80596900 -0.11878700

H 2.83918600 1.28476400 0.86715100

C 1.33797600 -0.62414600 0.49599000

H 1.21662300 -0.22286500 1.50368300

C 1.30359400 0.62831600 -0.50740200

O 4.41781800 1.69663400 -1.72902700

O 4.47835300 -1.48824300 1.78393300

C 0.33790700 -1.68519400 0.22033100

C -0.93072000 -1.52398700 0.78308400

C 0.59080800 -2.75312200 -0.67832400

C -1.96004000 -2.37912500 0.44129100

H -1.09863300 -0.70267000 1.46740400

C -0.39734100 -3.63051700 -1.00738200

H 1.58008200 -2.87266300 -1.10165400

C -1.72898000 -3.49737800 -0.47615200

H -0.22000400 -4.45791300 -1.68334500

C 0.24983800 1.65232000 -0.24766700

C -1.03625800 1.38301800 -0.73496600

C 0.45960700 2.77860200 0.53384300

C -2.09214900 2.21778900 -0.41915400

H -1.19765300 0.50460400 -1.34750600

C -0.59944600 3.63474600 0.83342400

H 1.44652000 3.00592200 0.91734900

C -1.86924100 3.35455500 0.37124700

H -0.44664700 4.52231600 1.43494700

O -3.38652300 2.03465600 -0.81167900

O -2.91008800 4.19004700 0.68205600

H -3.72072400 3.83528900 0.28812600

O -2.65576700 -4.28344100 -0.77244700

O -3.21344900 -2.27981100 0.88149900

C -3.53626200 -1.19131800 1.75226000

H -4.59884800 -1.28172600 1.95621900

H -2.96807100 -1.26835100 2.68023300

H -3.32841100 -0.23750000 1.26228200

C -3.67103100 0.87621100 -1.59126600

H -3.13216400 0.91081300 -2.54013400

H -3.40316900 -0.03389700 -1.04793400

H -4.74169300 0.89206200 -1.77529000

H 5.72629700 -0.43050600 -0.63607900

H 5.67648900 0.71366400 0.71393800

H 2.94005500 -1.18434800 -0.85395800

H 1.22823700 0.22477800 -1.51944500

46

15, E(SCF) = -1262.84491712, E(ZPE) = -1262.480650, E(G) = -1262.534099 a.u.

C -4.97443800 -0.30033300 0.10873900

C -4.12167900 -1.27422700 -0.60607400

C -4.22657800 0.73024000 0.58700900

C -2.83344600 0.59392800 0.05132500

C -2.72633000 -0.91072100 -0.13691100

H -2.69532500 -1.32837900 0.87561200

C -1.34994700 0.56822000 0.46957700

H -1.21762300 0.16958400 1.47582900

C -1.25585400 -0.69153500 -0.54315200

O -4.49437700 -2.16983200 -1.34598800

O -4.60779300 1.79107000 1.29434700

C -0.38970000 1.66768400 0.20160900

C 0.88431800 1.54593600 0.76439200

C -0.68028800 2.74172200 -0.67860200

C 1.88327000 2.44011500 0.43546400

H 1.08063100 0.72263300 1.43881900

C 0.27782100 3.65612800 -0.99610300

H -1.67525900 2.83821600 -1.09436900

C 1.61445200 3.56032200 -0.46913300

H 0.07044900 4.48858300 -1.65723200

C -0.17245400 -1.67969700 -0.26742300

C 1.11648700 -1.36260700 -0.71808200

C -0.36462600 -2.82862400 0.48557700

C 2.18931200 -2.17280800 -0.39629800

H 1.26768900 -0.46969200 -1.31157900

C 0.71268000 -3.65955600 0.79198300

H -1.35378800 -3.09548200 0.83661800

C 1.98371300 -3.33234200 0.36515200

H 0.57278000 -4.56549000 1.36874800

O 3.48562200 -1.94475300 -0.75844400

O 3.04209300 -4.14396200 0.68056100

H 3.84826200 -3.76340300 0.30186600

O 2.51341100 4.38195500 -0.75523800

O 3.13817000 2.38240400 0.88021600

C 3.49376000 1.30683300 1.75384300

H 4.55031400 1.43794600 1.96703600

H 2.91491000 1.36243300 2.67679900

H 3.32697800 0.34486700 1.26418300

C 3.74709300 -0.78639100 -1.54592800

H 3.22568000 -0.84864400 -2.50321500

H 3.44311200 0.12143000 -1.01825400

H 4.82065200 -0.77160500 -1.71242200

H -2.86408600 1.03822300 -0.94826900

H -1.17390500 -0.28315600 -1.55163300

H -6.04921300 -0.39233400 0.18397000

H -5.55465900 1.74549500 1.50208200

46

16, E(SCF) = -1262.90231898, E(ZPE) = -1262.543341, E(G) = -1262.599065 a.u.

C -2.48826900 3.86792200 -0.02221400

C -1.27557800 3.29341700 -0.70459700

C -3.29684400 2.67132200 0.41531500

C -2.53604500 1.43113000 0.13491900

C -1.19384500 1.77826600 -0.46005000

H -1.04159200 1.30195900 -1.43342600

C -3.08333400 0.23803600 0.42141800

H -4.08052500 0.28493800 0.85416600

C -0.05140200 1.49566000 0.46722800

H -0.26342500 1.53174100 1.52944400

O -0.46506000 3.91654300 -1.34768000

O -4.40315300 2.73713200 0.91972900

C -2.57900900 -1.11645100 0.25211400

C -1.32186200 -1.43444400 -0.29914300

C -3.41437100 -2.15934200 0.66268300

C -0.92936800 -2.75308700 -0.41040500

H -0.64637300 -0.66005200 -0.62813000

C -3.02293100 -3.48466800 0.54470800

H -4.38416600 -1.92652000 1.08523000

C -1.78703500 -3.78649600 0.00564000

H -3.66961800 -4.29277900 0.86181100

C 1.25806700 1.18258700 0.04557500

C 2.22359400 0.81944300 1.02665900

C 1.64663600 1.14956400 -1.31200900

C 3.48335000 0.41795700 0.65362100

H 1.93943700 0.84649300 2.07069000

C 2.91954900 0.74252800 -1.67659900

H 0.94900100 1.43835900 -2.08676200

C 3.83685400 0.36753700 -0.71067400

H 3.21621900 0.70802900 -2.71774700

O 4.48185700 0.03645100 1.50032700

O 5.08533900 -0.04309100 -1.07735500

H 5.58866800 -0.25922900 -0.27748400

O -1.39700900 -5.08381700 -0.11470900

H -0.51369500 -5.10972000 -0.51373000

O 0.26088000 -3.18534400 -0.91403100

C 1.20594300 -2.18964000 -1.29878800

H 2.08537600 -2.72585100 -1.64596500

H 0.80633300 -1.57261900 -2.10580200

H 1.46703400 -1.56295300 -0.44280000

C 4.20712200 0.08769000 2.89679500

H 3.95971000 1.10630600 3.20203700

H 3.38772800 -0.58694000 3.15248800

H 5.11746600 -0.23499300 3.39447800

H -2.17513200 4.44403000 0.85527800

H -3.05262000 4.53322700 -0.67720200

46

17, E(SCF) = -1262.90414826, E(ZPE) = -1262.544580, E(G) = -1262.599957 a.u.

C 2.90296900 -3.54321200 -0.04517300

C 1.57629500 -3.24396400 -0.51511300

C 3.51702200 -2.37842700 0.29038700

C 2.67249100 -1.20417400 0.07545600

C 1.34270400 -1.72021200 -0.42390300

H 1.13974600 -1.36937100 -1.44037200

C 3.10477900 0.03837000 0.33459800

H 4.11520000 0.11467000 0.72688100

C 0.20709700 -1.41964600 0.49629300

H 0.44279300 -1.31604900 1.54947400

O 0.72845200 -4.03019100 -0.92125800

O 4.74891300 -2.21198300 0.76578200

C 2.43766000 1.32940700 0.18616900

C 1.18812500 1.51169600 -0.43554900

C 3.10358900 2.44958200 0.68863200

C 0.63178900 2.77401400 -0.51874400

H 0.65743800 0.67503300 -0.86279900

C 2.54889300 3.71867400 0.59889500

H 4.07080000 2.32300600 1.15974200

C 1.31608600 3.88587600 -0.00118300

H 3.06435600 4.58624500 0.99101700

C -1.13317000 -1.26249300 0.08567200

C -2.10716400 -0.87732200 1.04914200

C -1.55288200 -1.41889200 -1.25397600

C -3.40921400 -0.64213800 0.67666700

H -1.79770200 -0.75636200 2.07906900

C -2.86759400 -1.17945000 -1.61813500

H -0.84856300 -1.73001900 -2.01419700

C -3.79699900 -0.78577600 -0.67074200

H -3.19073900 -1.29337400 -2.64561100

O -4.41776900 -0.25703100 1.51074500

O -5.08867700 -0.54640700 -1.04490400

H -5.59906600 -0.28365100 -0.26412000

O 0.76691100 5.13070200 -0.09127600

H -0.09070800 5.06278700 -0.53785900

O -0.56977400 3.07272500 -1.09232300

C -1.39287700 1.97841300 -1.48888100

H -2.31380300 2.41422600 -1.86753200

H -0.91093400 1.39727600 -2.27739600

H -1.60948000 1.33812600 -0.63097700

C -4.10224900 -0.11223100 2.89153300

H -3.74773300 -1.05764000 3.30658700

H -3.34740300 0.66324300 3.03559300

H -5.02623400 0.18146500 3.38215400

H 5.19544300 -3.06629700 0.86600000

H 3.32803900 -4.53441000 0.01794100

48

18, E(SCF) = -1413.25925355, E(ZPE) = -1412.889055, E(G) = -1412.946150 a.u.

C 2.57210600 -3.66409900 -0.42219800

C 1.36123600 -3.06336000 -1.08435500

C 3.34181600 -2.47999300 0.11128500

C 2.52086600 -1.25670200 -0.04631200

C 1.17007900 -1.62396900 -0.59861500

H 0.89947200 -0.99426700 -1.44492000

C 3.05472900 -0.05078500 0.21616200

H 4.08517800 -0.07452300 0.56409400

C 0.01722400 -1.64467900 0.44997700

O 0.63028100 -3.61794700 -1.86701100

O 4.46130700 -2.53753000 0.58303900

C 2.50238300 1.28856000 0.09915200

C 1.18031900 1.57589800 -0.29562600

C 3.35875400 2.35466400 0.39314100

C 0.75313900 2.88406000 -0.39202400

H 0.47529700 0.79003200 -0.51396400

C 2.92923800 3.66976100 0.30099400

H 4.37728600 2.14658300 0.69715700

C 1.63217900 3.93983100 -0.09278200

H 3.59172100 4.49507300 0.52832600

C -1.33846800 -1.22192900 -0.06333600

C -2.31809400 -0.91288400 0.89113800

C -1.64611300 -1.13642100 -1.41172800

C -3.56844200 -0.48717800 0.48753200

H -2.08754600 -0.99761800 1.94458400

C -2.91278400 -0.71932900 -1.81848800

H -0.92250400 -1.39315100 -2.17306000

C -3.86410900 -0.37972600 -0.88101100

H -3.15958500 -0.64225300 -2.86985300

O -4.59168900 -0.13647800 1.31522500

O -5.09455100 0.05654600 -1.28177900

H -5.62585300 0.25330300 -0.49542700

O 1.20617100 5.22662400 -0.18763900

H 0.27943400 5.23175500 -0.47315400

O -0.49637300 3.28210800 -0.76174900

C -1.41833200 2.26258600 -1.14214700

H -2.33550000 2.77408300 -1.42196200

H -1.03263200 1.70050300 -1.99508300

H -1.60948800 1.58688200 -0.30492300

C -4.35370000 -0.21028400 2.71810300

H -4.12447400 -1.23537700 3.01532800

H -3.53456900 0.45278800 3.00325400

H -5.27366000 0.11445800 3.19618500

H 2.23777100 -4.28276000 0.41959800

H 3.16006000 -4.29036600 -1.09251200

H -0.03454700 -2.62810300 0.92315300

O 0.33453100 -0.73045100 1.54324100

O 1.14907100 -1.26176600 2.40417400

48

19, E(SCF) = -1413.26744148, E(ZPE) = -1412.896054, E(G) = -1412.953723 a.u.

C -0.05722600 3.24106400 0.34328600

C -0.67896900 2.17735100 1.22258500

C 0.71225900 2.46820600 -0.71040600

C 0.65619500 1.06874100 -0.38511300

C -0.05341000 0.82628900 0.89161400

H 0.69213200 0.62677200 1.67770800

C 1.09893800 -0.08446800 -1.21321300

H 1.13394500 0.20590800 -2.26705600

C -0.96873500 -0.40947300 0.82805600

H -1.08892900 -0.82416300 1.82805300

O -1.53036800 2.35168100 2.05815800

O 1.27874200 2.95051200 -1.68761100

C 2.42948400 -0.67923000 -0.78546500

C 3.37861600 0.11492400 -0.13910700

C 2.71900300 -2.00323400 -1.07995600

C 4.60881600 -0.42291900 0.20338500

H 3.16278100 1.15055000 0.09227900

C 3.95009800 -2.54537600 -0.72630500

H 1.98558400 -2.62208700 -1.57993000

C 4.89341700 -1.76244700 -0.09011000

H 4.18754500 -3.57923700 -0.94439600

C -2.32821700 -0.20407600 0.21013600

C -3.39546900 -0.93397100 0.74354800

C -2.55322100 0.64926100 -0.86156900

C -4.66320100 -0.81395000 0.20106300

H -3.21838600 -1.59387600 1.58330400

C -3.82734100 0.77593700 -1.40612100

H -1.74749000 1.22181400 -1.30528900

C -4.87867200 0.05187800 -0.88024200

H -4.01430200 1.43859900 -2.24162100

O -5.77667900 -1.47105500 0.63261600

O -6.12823900 0.18375500 -1.41689900

H -6.73723500 -0.39702600 -0.93695300

O 6.10379700 -2.29939300 0.25563300

H 6.63555700 -1.61375900 0.68683000

O 5.61715400 0.24759400 0.83004200

C 5.38417500 1.60980200 1.17574100

H 6.29172000 1.95522700 1.66304000

H 5.19401800 2.20773900 0.28218400

H 4.54095000 1.69186600 1.86455900

C -5.62095200 -2.37870900 1.71930100

H -4.91705700 -3.17186600 1.45992300

H -5.27878000 -1.85376200 2.61331700

H -6.60431900 -2.80528700 1.89688300

H -0.81411700 3.89261700 -0.09618700

H 0.62090000 3.85998600 0.93901200

O 0.03736100 -1.05365500 -1.18530500

O -0.20902400 -1.44239000 0.16557700

48

20, E(SCF) = -1413.23469375, E(ZPE) = -1412.865508, E(G) = -1412.926840 a.u.

C 0.14347300 3.43964900 -0.87756500

C 1.22477200 2.43570700 -0.57816000

C -1.13905600 2.75910400 -0.48426400

C -0.84381300 1.31854100 -0.01907400

C 0.59813700 1.04744600 -0.47917000

H 0.56836300 0.65709500 -1.50316500

C -1.86304700 0.35020600 -0.50998900

C 1.38774200 0.11502600 0.42324700

O 2.39213800 2.69310700 -0.42070200

O -2.23706100 3.25082500 -0.49908700

C -3.06067200 0.09819300 0.05787700

C -3.96391000 -0.84942000 -0.57742700

C -3.50054200 0.74577000 1.28287400

C -5.18321600 -1.09798300 -0.06615800

H -3.62097700 -1.33147100 -1.48423400

C -4.71783400 0.51080200 1.79581100

H -2.83630400 1.44564700 1.77427900

C -5.64057300 -0.43402700 1.17526700

H -5.05842400 0.98929600 2.70560700

C 2.75242600 -0.28323700 -0.06081000

C 3.76699200 -0.42742700 0.89103800

C 3.02113500 -0.53131500 -1.39952700

C 5.03579500 -0.81290100 0.49692400

H 3.55202700 -0.22782600 1.93312500

C 4.29675700 -0.92004400 -1.79646000

H 2.25054500 -0.42150400 -2.15111000

C 5.30016700 -1.05971200 -0.85806000

H 4.52264000 -1.11419700 -2.83741200

O 6.10709500 -0.98189200 1.32246500

O 6.55278300 -1.43585500 -1.25224100

H 7.12420500 -1.48280300 -0.47073700

O -6.74756500 -0.67717300 1.65221600

O -6.10557500 -1.94883300 -0.57198800

C -5.74809300 -2.64377600 -1.76219500

H -6.59889900 -3.27074600 -2.01378500

H -5.55398500 -1.94044100 -2.57487700

H -4.86645200 -3.26559400 -1.59218900

C 5.90293700 -0.75734000 2.71397800

H 5.58734400 0.27197200 2.89492100

H 5.15940800 -1.45110000 3.11106400

H 6.86303500 -0.93466200 3.19074500

H -0.85685000 1.39387200 1.07557300

H 1.45157800 0.54038000 1.42515300

H -1.61815900 -0.16080500 -1.43780900

O 0.33272800 -1.77592400 -0.39803900

O 0.56788500 -1.09108400 0.67664800

H 0.29110000 4.39276300 -0.37033000

H 0.12292700 3.62517400 -1.95793000

48

21, E(SCF) = -1413.26835744, E(ZPE) = -1412.896523, E(G) = -1412.954244 a.u.

C -0.38091600 3.46957300 0.33743000

C 0.81988700 2.57692200 0.59291900

C -1.40568400 2.54959800 -0.30080500

C -0.95565500 1.12660000 -0.04013500

C 0.56780100 1.26398200 -0.10694900

H 0.84003900 1.43909300 -1.15651600

C -1.39623400 -0.01072500 -0.96433100

C 1.23771800 0.00651900 0.41357300

O 1.79122900 2.85905300 1.25099700

O -2.37361100 2.89632200 -0.93114400

C -2.60931600 -0.75471500 -0.46476000

C -3.78724100 -0.03508900 -0.28628200

C -2.55220800 -2.14076600 -0.18322100

C -4.92228900 -0.67623200 0.17619100

H -3.80300600 1.02194100 -0.51885500

C -3.65483300 -2.79469700 0.27820400

H -1.62578500 -2.67591700 -0.33949200

C -4.90218300 -2.10871400 0.48559100

H -3.63412200 -3.85411000 0.50201300

C 2.62403100 -0.23683500 -0.11761900

C 3.67160700 -0.41819000 0.78810700

C 2.87428000 -0.30151800 -1.48308400

C 4.95453200 -0.66118100 0.32507100

H 3.47326200 -0.36610600 1.85106600

C 4.15772700 -0.56464700 -1.94832800

H 2.07170200 -0.15848200 -2.19574700

C 5.19441800 -0.74421900 -1.05212400

H 4.36696700 -0.62707300 -3.00888500

O 6.05863400 -0.84174100 1.10610500

O 6.45675000 -0.99671200 -1.51481000

H 7.04800400 -1.11300500 -0.75610500

O -5.92961800 -2.68548400 0.90441000

O -6.09996100 -0.09148400 0.37523300

C -6.21992500 1.30186400 0.07374300

H -7.24830000 1.56491400 0.30120900

H -5.53815800 1.88445400 0.69510600

H -6.01199000 1.47778300 -0.98249200

C 5.88501300 -0.73350900 2.51535400

H 5.50990100 0.25668000 2.78140100

H 5.19965300 -1.50059900 2.88089800

H 6.86850400 -0.88298300 2.95238400

H -1.23293100 0.90854300 0.99982200

H 1.25365900 0.01897900 1.50633200

H -1.59202800 0.38021200 -1.96874900

O -0.27002300 -0.87349900 -1.15568400

O 0.33469100 -1.09636100 0.12282200

H -0.76665800 3.87399700 1.27701000

H -0.12664100 4.30680500 -0.31512800

48

22, E(SCF) = -1413.26352152, E(ZPE) = -1412.890528, E(G) = -1412.947132 a.u.

C 0.27531400 3.38668200 -0.52329100

C -0.96912600 2.63468700 -0.57605900

C 1.29548100 2.52272600 -0.31440700

C 0.83602800 1.09607100 -0.34452600

C -0.65261000 1.24711800 -0.05290200

H -0.76160900 1.33179300 1.03404700

C 1.38469800 0.04117700 0.61192300

C -1.38156100 0.01383700 -0.54038700

O -2.07217700 3.02725900 -0.92770400

O 2.59255400 2.76750200 -0.17424600

C 2.65660800 -0.60667600 0.14150200

C 3.86294700 -0.18945800 0.68481800

C 2.61444300 -1.60113100 -0.87181400

C 5.05140900 -0.74189000 0.22860800

H 3.86309400 0.56668500 1.45941400

C 3.76264200 -2.16775500 -1.32878400

H 1.65550600 -1.90627300 -1.27055500

C 5.04845700 -1.77342000 -0.81235400

H 3.75546400 -2.93129900 -2.09700400

C -2.69977900 -0.26039200 0.12969100

C -3.83200200 -0.44907000 -0.66670300

C -2.81065900 -0.34021300 1.51290400

C -5.05951200 -0.71006000 -0.08008600

H -3.74066500 -0.38610300 -1.74354400

C -4.03907200 -0.61718300 2.10228900

H -1.94244500 -0.19703400 2.14410800

C -5.15972900 -0.79737600 1.31394300

H -4.14103700 -0.68785800 3.17813200

O -6.23348700 -0.90268900 -0.74759100

O -6.36670900 -1.05978000 1.90229700

H -7.03808700 -1.15463900 1.21009000

O 6.12038000 -2.27165800 -1.21795500

O 6.26168900 -0.41728100 0.66862500

C 6.37112800 0.59083600 1.67886100

H 7.43365200 0.69863200 1.87344900

H 5.95833400 1.53289200 1.31593700

H 5.85344700 0.27419800 2.58493000

C -6.20093000 -0.81281500 -2.16855800

H -5.87798900 0.18090700 -2.48524300

H -5.53619200 -1.57065200 -2.58773600

H -7.21881900 -0.99130700 -2.50412100

H 0.96604000 0.74186900 -1.37290400

H -1.51655800 0.05488200 -1.62405400

H 1.52494100 0.47173900 1.60647200

O 0.32518200 -0.92723700 0.81145900

O -0.43892300 -1.09087400 -0.39067300

H 2.77271600 3.72098900 -0.19674700

H 0.35706900 4.45448200 -0.66636200

48

23, E(SCF) = -1413.27757115, E(ZPE) = -1412.906919, E(G) = -1412.965125 a.u.

C -0.10538500 4.04177400 0.44824200

C -1.40624600 3.52484800 0.80129800

C 0.63885900 3.03136500 -0.04596200

C -0.06977700 1.70527400 -0.09312300

C -1.43595700 2.02044100 0.53877100

H -1.55572600 1.51150400 1.49882900

C 0.70422200 0.66393800 0.67309400

C -2.62200400 1.63474000 -0.36007600

O -2.36447400 4.14969600 1.23557300

O 1.88996200 3.04694100 -0.48900600

C 1.66881700 -0.29410200 0.05571200

C 2.78112500 -0.75008300 0.90649800

C 1.87515300 -0.29190000 -1.40136800

C 3.99046500 -0.98512400 0.38333900

H 2.57630000 -0.83683700 1.96605600

C 3.07818000 -0.52871500 -1.92478100

H 1.01800600 -0.08986500 -2.03301500

C 4.23257100 -0.85358000 -1.07970100

H 3.25535600 -0.50929400 -2.99261600

C -2.68366200 0.14518300 -0.60785300

C -3.11473900 -0.69517600 0.42151900

C -2.23202300 -0.39352900 -1.80191200

C -3.09613100 -2.06689600 0.24229100

H -3.46134700 -0.26636000 1.35374200

C -2.21524700 -1.77491800 -1.98403800

H -1.88640200 0.25679900 -2.59628100

C -2.64190000 -2.60823700 -0.97046700

H -1.86361300 -2.21265700 -2.91012800

O -3.49715000 -2.99165800 1.16018300

O -2.62142800 -3.96545600 -1.14895900

H -2.95041400 -4.38934000 -0.34242900

O 5.34639500 -1.03382900 -1.54870600

O 5.09803800 -1.35965100 1.05768700

C 4.95275000 -1.54013600 2.46374200

H 5.92834800 -1.84370500 2.83219400

H 4.64812100 -0.60579000 2.94005500

H 4.21695300 -2.31941900 2.67340600

C -3.93734000 -2.50894600 2.42598700

H -4.81052100 -1.86408900 2.30964800

H -4.20523700 -3.38778400 3.00588800

H -3.13571100 -1.96441700 2.92894800

H -0.18177200 1.40653000 -1.13847700

H -3.54324100 1.95954900 0.16521100

H 0.90944900 0.92908300 1.70857900

O 0.35358600 -0.69198700 0.49109800

O -2.65540900 2.39102500 -1.51347900

H 2.27626000 3.93493800 -0.42380600

H 0.19865100 5.07317700 0.55180600

48

24, E(SCF) = -1413.30481965, E(ZPE) = -1412.934244, E(G) = -1412.992029 a.u.

C 0.21417300 4.08005400 -0.43185100

C 1.55732300 3.58206400 -0.55258900

C -0.64578500 3.00151800 -0.44437000

C 0.04109400 1.77120200 -0.55314000

C 1.51520400 2.04674300 -0.55024700

H 1.99831300 1.65369300 -1.44852100

C -0.65718300 0.57476500 -1.05538100

C 2.31337800 1.57166100 0.68952500

O 2.59698400 4.22968200 -0.60618400

O -1.98531900 3.01515100 -0.42286600

C -1.57150200 -0.27217600 -0.22214400

C -2.71523300 -0.89627100 -0.90430700

C -1.69790100 0.02481900 1.21282700

C -3.88534500 -1.04990100 -0.27199100

H -2.57029800 -1.17355800 -1.94063600

C -2.85956400 -0.13413000 1.84682500

H -0.81530800 0.39158000 1.72482400

C -4.04249000 -0.66455800 1.15802600

H -2.98051900 0.09855900 2.89722000

C 2.40435300 0.06474300 0.79278700

C 3.00198000 -0.64323600 -0.25634400

C 1.90229400 -0.62424100 1.88311100

C 3.06813400 -2.02315400 -0.21247700

H 3.41050900 -0.10716200 -1.10402700

C 1.96771300 -2.01736000 1.92880200

H 1.45051700 -0.09245800 2.71006000

C 2.54177600 -2.71583700 0.88916800

H 1.57622900 -2.56636200 2.77637500

O 3.62267200 -2.82034600 -1.17055600

O 2.60787800 -4.08389500 0.93633100

H 3.05155500 -4.40181700 0.13629400

O -5.11387400 -0.80299200 1.72825300

O -5.02024700 -1.55564100 -0.79645700

C -4.96886800 -1.94604900 -2.16627900

H -5.96026200 -2.31585700 -2.41150300

H -4.72328800 -1.08992500 -2.79835200

H -4.23094700 -2.73758500 -2.31191200

C 4.17845800 -2.17857700 -2.31375900

H 4.98852700 -1.50664100 -2.02359700

H 4.56895800 -2.97235700 -2.94459900

H 3.40921100 -1.62333000 -2.85432000

H 3.31891600 1.97928100 0.56455200

H -0.93602500 0.63413300 -2.10794600

O -0.27168200 -0.71721100 -0.64470900

O 1.80952600 2.17941500 1.86490900

H -2.31823900 3.92406900 -0.39037600

H -0.06368200 5.12233000 -0.37112300

H 0.88626300 1.91763000 1.99067200

48

25, E(SCF) = -1413.33752662, E(ZPE) = -1412.967371, E(G) = -1413.023157 a.u.

C -2.73439200 -2.57446700 0.86403000

C -1.85743600 -1.78891900 1.72379600

C -2.10396300 -2.80483900 -0.30074300

C -0.73789200 -2.18595400 -0.37904000

C -0.57968800 -1.60050000 1.00054600

C -0.72024900 -1.10412800 -1.53377100

C 0.49963700 -0.94689800 1.47156400

O -2.14252700 -1.38290800 2.85056100

O -2.52785500 -3.46149800 -1.38030400

C -1.61588000 0.07471500 -1.24035400

C -1.09642000 1.19376100 -0.58622600

C -2.96286600 0.02854200 -1.56428100

C -1.92928800 2.24711000 -0.25310600

H -0.04029500 1.22920000 -0.35094600

C -3.80373000 1.08878600 -1.23196600

H -3.36666700 -0.83590100 -2.07948100

C -3.29392500 2.18808300 -0.57112400

H -4.85801000 1.06752600 -1.47836900

C 1.78599200 -0.84665800 0.75448700

C 2.38290100 0.41472800 0.60453900

C 2.42897300 -1.98276700 0.27578600

C 3.59337200 0.52532000 -0.04929800

H 1.88149100 1.29682600 0.98289700

C 3.65767700 -1.87107400 -0.36198700

H 1.98425200 -2.95893500 0.41944200

C 4.23709100 -0.62734600 -0.53005600

H 4.17874100 -2.74595600 -0.72932700

O 4.26239800 1.68814900 -0.28126500

O 5.44064700 -0.52149400 -1.15706300

H 5.69916600 0.41255800 -1.18644000

O -4.12089400 3.22733000 -0.23602700

O -1.54347300 3.38635100 0.39303900

C -0.17433100 3.47915600 0.77428500

H -0.06235600 4.43915500 1.27104000

H 0.08459900 2.67230600 1.46459800

H 0.47070300 3.44213800 -0.10534600

C 3.57821300 2.90150000 0.02244900

H 2.65312200 2.96505800 -0.55360800

H 3.36038800 2.97036400 1.09009200

H 4.24949100 3.70565800 -0.26551600

H -0.00114900 -2.94258600 -0.65043900

O 0.42531900 -0.29529800 2.65724500

O 0.58031800 -0.78136400 -1.82133200

H -1.09108800 -1.65499100 -2.41658000

H -3.60330000 3.89378000 0.23977700

H 1.30623000 0.00505600 2.92465800

H -3.43294800 -3.78511200 -1.25412500

H -3.72663200 -2.89920300 1.14167500

48

26, E(SCF) = -1413.35879532, E(ZPE) = -1412.986298, E(G) = -1413.043952 a.u.

C 1.32250500 -0.55614100 2.67220900

C 0.43901600 -1.42000700 1.89065100

C 1.16291600 0.72437000 2.26932000

C 0.10434200 0.87569900 1.21430900

C -0.30464100 -0.53114100 1.00069900

C 0.50877200 1.29375900 -0.23013200

C -0.81734200 -0.70523100 -0.39145600

O 0.35200800 -2.64819100 1.94493400

O 1.77365500 1.82626500 2.69304900

C 1.97836700 1.12998400 -0.51178900

C 2.52989900 -0.14453300 -0.67040800

C 2.80402100 2.24342300 -0.52798300

C 3.89491000 -0.29058600 -0.83568100

H 1.88753200 -1.01481400 -0.68272700

C 4.17878500 2.09920900 -0.70085600

H 2.37860500 3.23247000 -0.40477700

C 4.72491200 0.84014900 -0.85007700

H 4.83427900 2.96090100 -0.72155000

C -2.33870400 -0.68818500 -0.42659500

C -3.01046500 0.53672800 -0.44856500

C -3.05315500 -1.87495600 -0.34715200

C -4.39295100 0.55992900 -0.41333800

H -2.44591600 1.45756000 -0.50390100

C -4.44427000 -1.84966400 -0.31657100

H -2.53430200 -2.82392900 -0.30948100

C -5.11330300 -0.64230000 -0.34772600

H -5.01876900 -2.76599500 -0.26564000

O -5.16627600 1.68213300 -0.43744000

O -6.47924100 -0.62047200 -0.31500400

H -6.77702500 0.30101600 -0.34938700

O 6.07564800 0.69923800 -1.02099100

O 4.54311500 -1.48114800 -0.98816300

C 3.74655400 -2.66187100 -0.94538500

H 4.43714000 -3.49368100 -1.05249600

H 3.21955800 -2.73719400 0.00918400

H 3.02636300 -2.67160500 -1.76631000

C -4.49476600 2.93779200 -0.48084100

H -3.85857700 3.06314300 0.39755900

H -3.89567600 3.02157800 -1.38957000

H -5.27354800 3.69535100 -0.48253300

H -0.70650800 1.51857100 1.57056400

O -0.29074700 -1.87727600 -0.94637200

O -0.31189400 0.45186500 -1.07032600

H 0.21281300 2.32263200 -0.42641600

H 6.28212500 -0.24170600 -1.12326000

H -0.73559300 -2.04641100 -1.78976200

H 2.43273800 1.61692600 3.37344200

H 2.01219900 -0.91270100 3.42376300

48

27, E(SCF) = -1413.36065553, E(ZPE) = -1412.988974, E(G) = -1413.045291 a.u.

C -4.96886600 -0.69130400 -0.64620300

C -4.41795900 0.64152700 -0.72546800

C -3.94665700 -1.57657300 -0.53486200

C -2.64299400 -0.91472300 -0.57146500

C -2.89599400 0.56825700 -0.55648300

H -2.39854600 1.08664500 -1.37768600

C -1.49954900 -1.57332700 -0.73042800

C -2.53593500 1.28099800 0.77231700

O -5.02929100 1.69386500 -0.85878100

O -3.99364100 -2.90214900 -0.44304400

C 0.84306700 -1.29699100 -0.55198700

C 1.95109400 -0.67946600 -1.14010100

C 0.96497200 -2.23908300 0.49031600

C 3.20827200 -0.98333300 -0.67536600

H 1.78107800 0.04049200 -1.92958800

C 2.20882100 -2.55353900 0.95186300

H 0.08218900 -2.67593700 0.93826900

C 3.39585900 -1.94338400 0.41699900

H 2.34174900 -3.26130600 1.76025300

C -1.04975900 1.26765000 1.06469700

C -0.19617500 1.97714800 0.21172000

C -0.51659300 0.56764800 2.13346400

C 1.16877700 1.95526200 0.42325100

H -0.61204100 2.52560000 -0.62493600

C 0.86216400 0.55704000 2.35652000

H -1.15721400 0.02140800 2.81359300

C 1.70307200 1.23526700 1.50423400

H 1.28925800 -0.00225300 3.17999900

O 2.09981000 2.57639700 -0.35575600

O 3.05872900 1.18371100 1.69140500

H 3.49187600 1.62585000 0.94505200

O 4.54132300 -2.20397800 0.84952800

O 4.34400300 -0.45709200 -1.13794800

C 4.25791600 0.45286800 -2.23706900

H 5.28016000 0.73787600 -2.46686900

H 3.80499900 -0.03966100 -3.09902900

H 3.67629500 1.33216600 -1.95716400

C 1.65064000 3.13799500 -1.58481500

H 0.95992900 3.96358900 -1.40348800

H 2.53795700 3.51178500 -2.08909800

H 1.16576200 2.37540200 -2.19957600

H -2.86053000 2.31686200 0.65238900

H -1.42368400 -2.65438400 -0.74856200

O -0.35662600 -0.87902200 -1.01836600

O -3.31121500 0.77195500 1.84383900

H -4.90993800 -3.21711200 -0.47127000

H -6.02201300 -0.92853700 -0.68247700

H -3.11706900 -0.16761000 1.96636300

48

28, E(SCF) = -1413.30625378, E(ZPE) = -1412.935910, E(G) = -1412.995233 a.u.

C 1.16673300 3.58131100 -0.29454900

C 1.85005300 2.42355800 -0.80876400

C -0.14236700 3.26402800 -0.10513600

C -0.43474100 1.88727500 -0.47215400

C 0.87553800 1.23637100 -0.83004100

H 0.86252100 0.76497900 -1.81393900

C -1.67552800 1.40482300 -0.53478800

C 1.31873100 0.21692000 0.23398000

O 3.02382800 2.34639100 -1.15748100

O -1.12653200 4.04660300 0.32955700

C -3.19083400 -0.36767400 -0.79298400

C -3.79087800 -0.25570500 0.45785600

C -3.79144400 -1.04157500 -1.83397000

C -5.04260500 -0.82069700 0.65486200

H -3.27305700 0.25398200 1.25939700

C -5.04365400 -1.61274800 -1.62309000

H -3.29566600 -1.11625500 -2.79230400

C -5.67270800 -1.50241000 -0.39823000

H -5.54340100 -2.14531600 -2.42407900

C 2.63599500 -0.48189000 -0.07604500

C 3.64015100 -0.50805300 0.89534500

C 2.81676300 -1.12737500 -1.29378500

C 4.84020500 -1.13542900 0.61635200

H 3.47916100 -0.01691500 1.84601200

C 4.02163400 -1.76135600 -1.57068500

H 2.02948900 -1.12644400 -2.03688400

C 5.02892500 -1.76819900 -0.62430700

H 4.18917400 -2.25844700 -2.51774700

O 5.90944000 -1.20885900 1.45551300

O 6.21014200 -2.38943300 -0.89919700

H 6.79878500 -2.29894000 -0.13452500

O -6.90741600 -2.03828800 -0.13542700

O -5.72951600 -0.77687400 1.82729600

C -5.09719000 -0.13511900 2.93025600

H -5.79395200 -0.21493300 3.76013100

H -4.16168300 -0.63694700 3.18607200

H -4.90681200 0.91757600 2.71159600

C 5.77512500 -0.60087200 2.73715100

H 4.96593700 -1.06938900 3.30047400

H 6.72098100 -0.76082200 3.24694700

H 5.58726800 0.46973500 2.63565900

H 1.40741100 0.71656900 1.20599900

H -2.54222500 1.99682000 -0.25888000

O -1.92579900 0.16608200 -1.01703600

O 0.46799400 -0.87936100 0.31920000

H 1.62230200 4.54657600 -0.12908500

H -0.79928500 4.94200400 0.50407000

H -7.25149100 -2.46503200 -0.93194600

48

29, E(SCF) = -1413.34069755, E(ZPE) = -1412.968325, E(G) = -1413.026573 a.u.

C 2.00125000 4.17525300 -0.11814200

C 2.66755500 2.89246100 -0.11594800

C 0.64371200 3.97454600 -0.28644100

C 0.36073700 2.60872700 -0.48101500

C 1.61725100 1.81940000 -0.41089000

H 1.87325600 1.30868600 -1.34241600

C -0.75886200 1.76958700 0.01583400

C 1.29313300 0.80602100 0.71672900

O 3.85541800 2.66711300 0.07682100

O -0.33317800 4.88592700 -0.23130500

C -2.40809800 0.27313800 -0.86602000

C -2.93382500 0.02028300 0.40037300

C -3.00921500 -0.24089200 -2.00127700

C -4.09186300 -0.73911100 0.51470100

H -2.44760900 0.39313000 1.28869000

C -4.16155400 -1.00834600 -1.87338200

H -2.58098200 -0.03908000 -2.97412900

C -4.71125500 -1.25493900 -0.62973700

H -4.64592400 -1.41712900 -2.75286200

C 1.66631700 -0.61335600 0.37539900

C 3.02005700 -0.95699600 0.44547700

C 0.73568900 -1.54795400 -0.04386700

C 3.42901000 -2.22870800 0.08791700

H 3.74035300 -0.21891400 0.77814000

C 1.14824400 -2.83239000 -0.40023900

H -0.31432400 -1.28971900 -0.09414000

C 2.48241500 -3.17358400 -0.33727800

H 0.43298500 -3.57695600 -0.72750500

O 4.71426000 -2.68324500 0.11374500

O 2.88291800 -4.43738600 -0.68529200

H 3.84463600 -4.49734400 -0.58536000

O -5.85197900 -1.99773000 -0.44077100

O -4.68962100 -1.04246100 1.70024100

C -4.07063500 -0.56056600 2.88858500

H -4.69054800 -0.91002400 3.70982900

H -3.06150200 -0.96499100 2.99255700

H -4.03574600 0.53082800 2.89451200

C 5.71980200 -1.76967800 0.54153600

H 5.53617900 -1.44975300 1.56923400

H 6.66025200 -2.31111900 0.48758500

H 5.75557700 -0.89974500 -0.11728100

H 1.80467100 1.10431000 1.63431200

H -1.56415300 2.31172700 0.51103000

O -1.26152100 1.02012900 -1.06977800

O -0.12373000 0.94037600 0.97162900

H 2.48818900 5.12505200 0.05149700

H 0.03179100 5.76307000 -0.03989800

H -6.20683300 -2.26119800 -1.30003000

50

30, E(SCF) = -1414.45775797, E(ZPE) = -1414.068179, E(G) = -1414.131635 a.u.

C -0.10631400 -0.44098800 0.07899800

C -1.41594100 -1.02197200 -0.05038900

C 1.05653600 -1.20537500 0.04861600

C 2.30451600 -0.60155300 0.17233700

H 2.33897300 0.47005100 0.31293800

C -2.55360900 -0.09935500 -0.00474800

H -2.31566900 0.95131900 0.10748000

C 3.57980600 -1.37738000 0.14310800

H 3.47409900 -2.25286300 -0.50328400

C -3.81719400 -0.54012500 -0.09319900

H -3.97523000 -1.61112700 -0.19468400

O -1.56355000 -2.25863900 -0.19558500

O 1.01073100 -2.54671700 -0.10095300

C 4.75038900 -0.54568100 -0.30908900

C 5.11468700 0.58684200 0.42817500

C 5.46265100 -0.89122700 -1.44475200

C 6.18263800 1.36018000 0.01568100

H 4.55801500 0.84891200 1.31952400

C 6.53691800 -0.10900500 -1.86637100

H 5.18355300 -1.77306600 -2.00891600

C 6.89631000 1.00862100 -1.14245300

H 7.10331900 -0.36555800 -2.75276400

C -5.02788500 0.27296900 -0.06401300

C -6.26666100 -0.38679000 -0.11504500

C -5.00481600 1.66733100 0.00900700

C -7.44593800 0.33346500 -0.08461800

H -6.28625500 -1.46748900 -0.17197300

C -6.18679100 2.39020100 0.03642300

H -4.06443900 2.20139500 0.04495300

C -7.40475000 1.73316900 -0.01233700

H -6.18019200 3.47157900 0.08925800

O -8.70182800 -0.19608000 -0.12186500

O -8.56008500 2.45527300 0.01158500

H -9.31378300 1.84767000 -0.03475900

O 7.95073300 1.77741800 -1.55207500

H 8.06112000 2.50951700 -0.92693600

O 6.63994800 2.48335500 0.63859600

C 5.96395100 2.89929700 1.82135200

H 6.47598400 3.79676200 2.15744300

H 4.91837800 3.12899500 1.60708300

H 6.02575300 2.12750300 2.59105500

C -8.80948600 -1.61218800 -0.22781700

H -8.33694400 -1.96602500 -1.14615500

H -8.35423800 -2.09947000 0.63659800

H -9.87322600 -1.83141200 -0.25358700

H -0.01159500 0.62900400 0.20040200

H 0.05550400 -2.79055700 -0.17699300

O 3.74461800 -1.84916000 1.50005500

O 4.87592900 -2.71159900 1.53759100

H 4.49079700 -3.57856300 1.33133200

50

31, E(SCF) = -1414.48019271, E(ZPE) = -1414.092876, E(G) = -1414.157607 a.u.

C -0.13715700 -0.53726300 0.37358600

C -1.46068900 -1.08996200 0.19708000

C 0.94912600 -1.34748600 0.42174100

C 2.31750300 -0.80092100 0.55863000

H 2.36953200 0.24816200 0.82643600

C -2.58102100 -0.14595800 0.16692500

H -2.32859700 0.90261900 0.26503300

C 3.43613900 -1.40267500 -0.19738400

H 3.20586000 -2.26412300 -0.81520700

C -3.84610300 -0.56919200 0.03136400

H -4.01525600 -1.63939800 -0.05902300

O -1.63002200 -2.32489800 0.08302100

O 0.88580100 -2.67000700 0.32410400

C 4.63108700 -0.61951900 -0.59798100

C 5.06106100 0.47166600 0.17087500

C 5.35177700 -1.00213300 -1.71728900

C 6.20110200 1.16310900 -0.19568100

H 4.49787400 0.78682300 1.04036900

C 6.49558200 -0.29908500 -2.09197800

H 5.02614300 -1.85266500 -2.30357100

C 6.92412700 0.77078400 -1.33493500

H 7.06715300 -0.58654500 -2.96545200

C -5.04558300 0.25950800 -0.00772200

C -6.28549300 -0.38378300 -0.15306900

C -5.01055200 1.65150000 0.09582500

C -7.45496900 0.35161700 -0.19603200

H -6.31281300 -1.46287900 -0.23303200

C -6.18284900 2.38931300 0.05489600

H -4.06782000 2.17047500 0.20899400

C -7.40243300 1.74869500 -0.08671300

H -6.16814400 3.46889900 0.13544100

O -8.71092200 -0.16031000 -0.33651400

O -8.54836600 2.48523500 -0.12009300

H -9.30611900 1.88938800 -0.22126300

O 8.05049200 1.45415000 -1.69601700

H 8.20090700 2.16633400 -1.05630200

O 6.72448200 2.23241800 0.46250200

C 6.04705300 2.68083900 1.63334700

H 6.61931400 3.52655800 2.00418300

H 5.03163000 3.00033800 1.39113500

H 6.02024300 1.89045300 2.38625500

C -8.82630700 -1.57360900 -0.47023200

H -8.28907700 -1.92009700 -1.35537000

H -8.44263600 -2.07699700 0.41922000

H -9.88740900 -1.77926900 -0.58072800

H -0.00691800 0.53024500 0.47504000

H -0.08033600 -2.89282900 0.20806200

O 3.26222400 -1.64739800 1.20516100

O 5.82643700 -1.01087900 2.34584100

H 4.89702500 -1.26465200 2.15422400

50

32, E(SCF) = -1414.55364356, E(ZPE) = -1414.161362, E(G) = -1414.222347 a.u.

C 0.46593500 0.47033700 -0.47434900

C 1.61393300 -0.24216100 -0.03311500

C -0.81376500 -0.07604500 -0.35972400

C -1.89978100 0.65630300 -0.83502100

H -1.70211400 1.61646300 -1.29226800

C 2.91963500 0.39865200 -0.21341700

H 2.91163900 1.39327300 -0.64353200

C -3.69777100 -0.84469500 -0.19333200

C 4.05689900 -0.22313600 0.12895700

H 3.97903200 -1.22429300 0.54495300

O 1.51164400 -1.39284900 0.49099600

O -0.98070500 -1.29906900 0.19174700

C -5.19415900 -0.68241700 -0.12853700

C -5.72246400 0.54835200 0.27347400

C -6.03854900 -1.74535000 -0.40022500

C -7.09189100 0.70519300 0.37932900

H -5.05834500 1.37320400 0.49383900

C -7.41853000 -1.58713300 -0.28871200

H -5.63393100 -2.70063300 -0.70300900

C -7.94521300 -0.37296900 0.09822200

H -8.09278500 -2.40732600 -0.50086400

C 5.41576200 0.29476100 -0.00400700

C 6.47751800 -0.55028500 0.35746000

C 5.70178500 1.57734800 -0.47484600

C 7.78643400 -0.12010600 0.24510500

H 6.25706500 -1.54435100 0.72467100

C 7.01444000 2.00971900 -0.58764300

H 4.90237000 2.25037900 -0.75558000

C 8.05617100 1.16871300 -0.23486500

H 7.24709000 3.00240400 -0.95222100

O 8.89311300 -0.85018100 0.56483500

O 9.34401300 1.60254800 -0.35356800

H 9.94192500 0.89232200 -0.07544500

O -9.30001400 -0.22050300 0.20775900

H -9.48928000 0.68889000 0.48317900

O -7.73220800 1.85149700 0.74711800

C -6.92510300 2.98762300 1.04038200

H -7.61404600 3.78672400 1.29973700

H -6.26273100 2.78266300 1.88360000

H -6.33762100 3.27761500 0.16705400

C 8.68586700 -2.19181400 0.99572100

H 8.10829000 -2.21436200 1.92173200

H 8.17333900 -2.76804300 0.22293700

H 9.67429300 -2.60838500 1.16883300

H 0.58008900 1.44982500 -0.91642500

H -0.05070200 -1.61346400 0.42498500

O -3.19271200 0.35187200 -0.84296200

H -3.25788700 -0.87416100 0.80461000

O -3.35673400 -1.97220500 -0.92700100

H -2.44977800 -2.21297300 -0.67731700

49

33, E(SCF) = -1339.32832942, E(ZPE) = -1338.942102, E(G) = -1339.002654 a.u.

C -0.08440700 3.59290700 -0.89457200

C -1.42259100 3.12777100 -0.30419800

C 1.02432000 2.96448500 -0.09438600

C 1.72025400 1.85482500 -0.48962200

H 1.45082400 1.45702200 -1.46117000

C -1.77620300 1.72638400 -0.50179800

H -1.08928800 1.12589500 -1.08583500

C 2.72244900 1.24912600 0.26864800

H 2.97584200 1.69001400 1.22654600

C -2.92234300 1.23521000 -0.00560900

H -3.55549600 1.91000900 0.56551200

O -2.10138800 3.94373100 0.30252600

O 1.26405400 3.53207300 1.11878300

C 3.47291000 0.08456700 -0.11745800

C 4.50868900 -0.36385300 0.73290000

C 3.22797000 -0.63557800 -1.29810000

C 5.26265700 -1.47202100 0.40550900

H 4.70559300 0.18219700 1.64631800

C 3.98719100 -1.74957300 -1.62194500

H 2.43846000 -0.33009100 -1.97187200

C 5.00153000 -2.17372500 -0.78175400

H 3.79798900 -2.30565300 -2.53194700

C -3.42788300 -0.12288000 -0.14908400

C -4.67335400 -0.42253000 0.42700600

C -2.73407700 -1.12354400 -0.83272400

C -5.20701100 -1.69185800 0.31568000

H -5.20876000 0.35489400 0.95661100

C -3.26851700 -2.39730700 -0.94328900

H -1.77161900 -0.91610000 -1.28253400

C -4.49865300 -2.68580800 -0.37570000

H -2.74160500 -3.18315400 -1.46965900

O -6.40281700 -2.10065000 0.82547700

O -5.01626800 -3.93947500 -0.49290700

H -5.87432600 -3.97055200 -0.04273800

O 5.74535000 -3.27406000 -1.11135000

H 6.40368100 -3.42355800 -0.41634500

O 6.28678400 -1.98342700 1.15047200

C 6.60855100 -1.31135600 2.36391500

H 7.43848200 -1.85977600 2.80110400

H 6.91051000 -0.28108900 2.16501900

H 5.75771700 -1.32274400 3.04792100

C -7.16000500 -1.14602900 1.56361700

H -6.60115200 -0.80624600 2.43782900

H -7.42386100 -0.29349900 0.93505300

H -8.06231600 -1.66133900 1.88100100

H -0.02024500 3.24853100 -1.92918000

H 0.86795600 4.41791000 1.13522200

O 0.00321900 4.99958200 -0.82298300

H -0.79872400 5.30986200 -0.36922400

50

34, E(SCF) = -1414.45552863, E(ZPE) = -1414.067543, E(G) = -1414.128594 a.u.

C 0.09084400 3.21382000 -0.79966700

C -0.91582800 2.52163900 0.06998900

C 1.53079000 2.84946500 -0.41403300

C 1.87229800 1.43213200 -0.51822200

H 1.11104000 0.76894400 -0.91173900

C -1.80829300 1.60380000 -0.42093800

H -1.75935400 1.42159600 -1.48792100

C 3.08681500 0.99935400 -0.14385500

H 3.78253200 1.73508300 0.25227600

C -2.73547600 0.92211800 0.36485500

H -2.76714300 1.14185400 1.42611300

O -0.86120500 2.75796700 1.41501100

O 2.30492200 3.72513500 -0.05837400

C 3.59036300 -0.36501600 -0.20560800

C 4.89116500 -0.60512500 0.26662700

C 2.84363200 -1.42837700 -0.71698800

C 5.42516600 -1.87860100 0.22761300

H 5.46877800 0.22129000 0.66037300

C 3.37838800 -2.70588800 -0.75585500

H 1.84029700 -1.26633400 -1.08915400

C 4.66173200 -2.93689600 -0.28795200

H 2.81134500 -3.53948000 -1.15057800

C -3.67774100 -0.05506200 -0.11256800

C -4.58487200 -0.62109900 0.81186700

C -3.74279800 -0.47955800 -1.44971500

C -5.50826100 -1.56306800 0.40866700

H -4.54399800 -0.30091800 1.84500900

C -4.67092300 -1.42805000 -1.84955100

H -3.06349900 -0.07335100 -2.18746000

C -5.55165700 -1.97444700 -0.93258400

H -4.72043500 -1.75968400 -2.87941300

O -6.42868600 -2.16964600 1.21396300

O -6.46207200 -2.91180800 -1.33687400

H -6.98304700 -3.18955900 -0.56831900

O 5.17593000 -4.19600900 -0.33409800

H 6.07026200 -4.18751800 0.03896500

O 6.67068500 -2.23252700 0.65299800

C 7.49714100 -1.20054300 1.18424100

H 8.43388900 -1.67847100 1.45709600

H 7.67990800 -0.43008000 0.43286100

H 7.03705300 -0.75670700 2.06922800

C -6.44153200 -1.79642400 2.58790300

H -5.48455100 -2.02804300 3.05984900

H -6.66087400 -0.73232600 2.69639800

H -7.23055400 -2.38234300 3.05142000

H -0.06588100 2.89153200 -1.83255400

H 1.02445600 5.23083500 0.54668900

O -0.06963000 4.62962500 -0.86208100

O 0.05613500 5.17998800 0.44924500

H -0.45457100 3.62682100 1.56718500

50

35, E(SCF) = -1414.45482650, E(ZPE) = -1414.069224, E(G) = -1414.132133 a.u.

C -0.00460800 0.13064100 -1.26711900

C -1.27252000 -0.59451000 -0.84366600

C 1.25365100 -0.64254800 -0.84399700

C 2.46617100 0.12137200 -0.62663200

H 2.39074600 1.19573700 -0.71356200

C -2.47352900 0.13046100 -0.68660400

H -2.41513700 1.19619000 -0.84758600

C 3.61778100 -0.51195600 -0.33864200

H 3.60071900 -1.59764900 -0.28299200

C -3.62361700 -0.50580600 -0.33955500

H -3.58279100 -1.58088100 -0.18500500

O -1.24156400 -1.86703400 -0.68621600

O 1.20560500 -1.87758700 -0.75633300

C 4.90868700 0.10616600 -0.08882800

C 6.01270600 -0.73620800 0.12805400

C 5.08596200 1.49098200 -0.04548100

C 7.26089400 -0.19888000 0.37339900

H 5.87068200 -1.80871600 0.09625900

C 6.33588700 2.02973100 0.20798200

H 4.24781600 2.15659200 -0.20486700

C 7.42067900 1.19442100 0.42105700

H 6.48813800 3.10069600 0.25045000

C -4.91325300 0.09978500 -0.14492800

C -5.98737500 -0.73952600 0.21406800

C -5.13356400 1.47536800 -0.29407000

C -7.24208600 -0.20921900 0.41876000

H -5.80965100 -1.80095800 0.32978600

C -6.39274700 2.00564500 -0.09327000

H -4.31984800 2.13522400 -0.56397100

C -7.44702600 1.17316100 0.25704600

H -6.58286400 3.06524600 -0.20548800

O -8.35667900 -0.90383600 0.77369100

O -8.67577100 1.70333600 0.44743900

H -9.29931900 0.99630000 0.67641100

O 8.64250700 1.73460700 0.67422100

H 9.28230100 1.02031500 0.81763600

O 8.40544100 -0.90608400 0.58940200

C 8.31286100 -2.32714400 0.54390300

H 9.31562900 -2.69928800 0.73440000

H 7.63013400 -2.69292800 1.31320300

H 7.97619500 -2.65860000 -0.44030800

C -8.21387800 -2.30871500 0.96861800

H -7.89107900 -2.79244600 0.04475300

H -7.49764900 -2.51290800 1.76672500

H -9.19691900 -2.67322700 1.25260000

H -0.00815600 0.03474400 -2.37414000

H 0.85024700 1.22472100 1.23626200

O -0.00242100 1.44457700 -0.89978500

O -0.05504000 0.89094100 1.17354000

H -0.25227700 -2.15883800 -0.74881700

50

36, E(SCF) = -1414.51254498, E(ZPE) = -1414.124240, E(G) = -1414.187360 a.u.

C -0.12566000 -0.54779300 0.67044100

C -1.26316000 -1.59589800 0.57632000

C 1.32231600 -1.14738100 0.20006600

C 2.40074800 -0.18663500 0.30995900

H 2.14456600 0.79422700 0.68560600

C -2.56389500 -0.89035500 0.86083500

H -2.63307200 -0.50266700 1.87302600

C 3.64426600 -0.54294800 -0.06359200

H 3.79221000 -1.55880700 -0.42175500

C -3.54110300 -0.73459200 -0.02711400

H -3.40071400 -1.12786600 -1.02923700

O -1.32184300 -2.19639400 -0.68104100

O 1.37540600 -2.27900900 -0.20220800

C 4.83275000 0.28895900 -0.03460500

C 6.04919200 -0.29224700 -0.43351200

C 4.81264600 1.62629800 0.36957200

C 7.21270300 0.45043000 -0.42170300

H 6.06038800 -1.32831700 -0.74644700

C 5.97943300 2.37112800 0.38105400

H 3.88686300 2.09461200 0.67729100

C 7.17627900 1.79248300 -0.01118100

H 5.98043300 3.40819900 0.69174000

C -4.83223100 -0.06803600 0.20535800

C -5.70103200 0.07389900 -0.88560500

C -5.23046000 0.41467700 1.45091700

C -6.92985300 0.69225200 -0.73173400

H -5.39635600 -0.30241300 -1.85367100

C -6.46335800 1.03433800 1.60689100

H -4.58345200 0.31173900 2.31271500

C -7.31460800 1.17270200 0.52571100

H -6.78237400 1.40912400 2.57158700

O -7.84936500 0.88722600 -1.72125700

O -8.52901300 1.78037100 0.69181500

H -8.99385600 1.78789700 -0.15809400

O 8.31446700 2.53341600 0.00415200

H 9.05650700 1.98362700 -0.29170300

O 8.44753300 0.00264500 -0.78182700

C 8.55737700 -1.36171600 -1.17818200

H 9.60645700 -1.52278000 -1.40989200

H 8.25237300 -2.02276300 -0.36464300

H 7.94937600 -1.55554900 -2.06388400

C -7.52148200 0.41102300 -3.02275100

H -6.62456600 0.90636500 -3.39978300

H -7.37181200 -0.67037700 -3.01128000

H -8.37029300 0.65801500 -3.65442600

H -0.25958700 0.25913300 -0.07256700

H -0.78670800 -2.17949400 2.37230500

O 0.09081900 -0.11479100 1.90552300

O -1.03572100 -2.60989800 1.54022100

H -0.46984200 -2.63397000 -0.83276700

49

37, E(SCF) = -1413.97896585, E(ZPE) = -1413.602018, E(G) = -1413.664001 a.u.

C -0.25938000 -0.25424400 1.64753000

C -0.91641800 0.30534800 -1.33786100

C 1.01052200 0.55277800 1.35233300

C 2.20857500 -0.18879500 0.99977900

H 2.12045700 -1.26631900 0.94882400

C -2.18325200 -0.37566900 -1.05847100

H -2.15595900 -1.45723800 -1.04306400

C 3.35730100 0.46619300 0.75295200

H 3.34537600 1.55055400 0.83632300

C -3.28973800 0.33983800 -0.82258200

H -3.20867900 1.42347400 -0.86012600

O -0.74046800 1.50703600 -1.39430600

O 0.90886900 1.76615400 1.44212700

C 4.63635400 -0.11638100 0.38522900

C 5.72721800 0.75223900 0.20852900

C 4.81887400 -1.48900900 0.20102300

C 6.96640500 0.25322200 -0.13947400

H 5.58198100 1.81534900 0.35084300

C 6.06158800 -1.98968500 -0.14915400

H 3.99231100 -2.17549700 0.32900100

C 7.13337400 -1.12837300 -0.32105900

H 6.21802900 -3.05064500 -0.29756600

C -4.61509600 -0.18193400 -0.50867800

C -5.65790200 0.74018900 -0.32460600

C -4.87877300 -1.54557600 -0.37360600

C -6.93217600 0.30048400 -0.02049300

H -5.45131200 1.79764000 -0.42800100

C -6.15577900 -1.98672800 -0.06467300

H -4.08945100 -2.27358100 -0.50810900

C -7.18044500 -1.07296900 0.11630600

H -6.37256300 -3.04170200 0.04596900

O -8.02350700 1.09444700 0.17386900

O -8.43145700 -1.51492800 0.42396900

H -9.01803300 -0.75031500 0.52828800

O 8.34847300 -1.63015800 -0.66788300

H 8.98302000 -0.90085900 -0.74404000

O 8.09516400 0.99023700 -0.33804900

C 7.99476800 2.39939100 -0.15163700

H 8.98573000 2.79929800 -0.34775400

H 7.27703900 2.82924100 -0.85286300

H 7.69913700 2.63057200 0.87353900

C -7.83869700 2.50093000 0.04393500

H -7.11887400 2.86446800 0.77975800

H -7.50048500 2.75218500 -0.96323500

H -8.81096000 2.94972500 0.22756800

H -1.14687000 0.36548300 1.85368000

O -0.28631000 -1.45623600 1.66545300

O 0.09293600 -0.56589600 -1.51338900

H 0.91655700 -0.07457900 -1.67529700

46

TS11-12, E(SCF) = -1252.07714973, E(ZPE) = nofreq, E(G) = nofreq a.u.

C 4.97902400 -0.29203300 -0.02844100

C 4.05796800 -1.48434300 0.25628600

C 4.19119800 0.98957600 -0.28306500

C 2.89836500 1.08758100 0.43396700

C 2.69420100 -1.26220600 -0.27692400

H 2.72066100 -0.78730700 -1.25395800

C 1.75837600 1.57044000 -0.12019600

H 1.75507800 1.65204000 -1.20454900

C 1.50384000 -1.80457700 0.07843700

O 4.40977800 -2.46770800 0.87687100

O 4.54917500 1.79979200 -1.11410800

C 0.49799300 1.58995100 0.54515400

C -0.63319000 1.37953100 -0.26734800

C 0.34724100 1.62577400 1.96490200

C -1.83007100 1.03370500 0.29005600

H -0.48512600 1.34230000 -1.33680600

C -0.86066400 1.40623100 2.53798300

H 1.20193300 1.84685200 2.59113400

C -2.01584900 1.04745800 1.74496600

H -0.99530400 1.44503400 3.61133600

C 0.26284100 -1.31709400 -0.49787400

C -0.97095100 -1.66228700 0.09091800

C 0.25822500 -0.38713300 -1.55523600

C -2.13455600 -1.04766500 -0.32687700

H -0.98592800 -2.38569200 0.89727000

C -0.90626500 0.23502600 -1.96400900

H 1.18399200 -0.11559300 -2.04501400

C -2.10030500 -0.05980300 -1.33222500

H -0.90141700 0.97100500 -2.75947300

O -3.38577300 -1.29966100 0.12878500

O -3.23762700 0.56891500 -1.69281200

H -3.96688500 0.23361200 -1.14559600

O -3.11484600 0.75621300 2.25445200

O -2.91053400 0.60915900 -0.38792500

C -2.81478100 0.56606900 -1.81113800

H -3.81125300 0.32697300 -2.17303300

H -2.49458500 1.53239300 -2.20786500

H -2.11344300 -0.23011300 -2.10039200

C -3.57316300 -2.42135700 0.98067700

H -3.14447700 -3.31690300 0.52416200

H -3.12088800 -2.25103800 1.96008400

H -4.64738200 -2.53720400 1.09388500

H 5.70695500 -0.15437100 0.77305700

H 5.51906100 -0.52570000 -0.95040400

H 3.00589000 0.92677500 1.50330100

H 1.43485700 -2.46379500 0.94066000

46

TS12-14, E(SCF) = -1262.84005237, E(ZPE) = -1262.479947, E(G) = -1262.534058 a.u.

C -5.06499900 -0.14286800 0.02504400

C -4.10479300 -0.99901700 -0.81095400

C -4.17115600 0.80383200 0.83534100

C -2.82139800 0.71351000 0.17059500

C -2.75653700 -0.78288600 -0.17039900

H -2.79303800 -1.32553200 0.78255400

C -1.42079000 0.80512000 0.71072500

H -1.23460900 0.17226500 1.57370800

C -1.35557100 -0.75725400 -0.70664600

O -4.39516000 -1.64329900 -1.78722300

O -4.49906000 1.42307000 1.81621900

C -0.42076900 1.72874400 0.37833200

C 0.88405100 1.50792700 0.91545000

C -0.59906900 2.75694900 -0.59666900

C 1.95047400 2.23717400 0.48360100

H 1.00549800 0.72024300 1.64824800

C 0.44827800 3.51881300 -1.00271900

H -1.58471600 2.93446600 -1.00850300

C 1.78473500 3.30888800 -0.50022700

H 0.32078900 4.31134800 -1.73004300

C -0.29331400 -1.66551500 -0.38675200

C 0.99814800 -1.34137400 -0.85916200

C -0.45198900 -2.76133600 0.47138900

C 2.08864700 -2.08516300 -0.46403900

H 1.11819000 -0.48861000 -1.51597900

C 0.64362000 -3.52492100 0.84017600

H -1.43291800 -3.02641300 0.84404200

C 1.90984800 -3.18929200 0.38844000

H 0.53548900 -4.38264100 1.49205300

O 3.38210500 -1.84713400 -0.81815100

O 2.98101000 -3.93490000 0.77366600

H 3.78530000 -3.55850400 0.38489000

O 2.76016900 3.99139100 -0.87933700

O 3.22967300 2.06114500 0.88295700

C 3.49026900 0.97230000 1.76423300

H 4.56608400 0.95798500 1.91523700

H 2.98544200 1.12097400 2.72097700

H 3.16472300 0.02845800 1.31771300

C 3.64026500 -0.68088300 -1.59633200

H 3.16630200 -0.76300100 -2.57633700

H 3.28093600 0.21470800 -1.08311800

H 4.71917400 -0.63065600 -1.71360800

H -5.74145600 0.42362200 -0.61854700

H -5.66619400 -0.77178000 0.68510000

H -2.91742800 1.25415300 -0.77839300

H -1.21517400 -0.12974800 -1.58162000

29

TS1-2, E(SCF) = -990.085314714, E(ZPE) = -989.883390, E(G) = -989.928230 a.u.

C 4.79358700 -0.03869900 0.13813400

C 3.80584800 0.93483500 0.12969900

C 2.46771800 0.56415400 0.05254300

C 2.12318200 -0.79252100 -0.00961900

C 3.11773800 -1.76334900 0.00166900

C 4.45027300 -1.38511800 0.07684800

C 1.42764800 1.61880100 0.06342600

C 0.70779500 -1.20499500 -0.10596900

C -0.32280400 -0.17405400 -0.14677500

C -0.01791300 1.19752900 -0.07889700

C -0.98180600 2.19581400 -0.11390300

H -0.68366700 3.23054300 -0.02717200

C -2.30593800 1.85372800 -0.27765900

C -2.78032400 0.45774200 -0.23781300

C -1.66100000 -0.55966000 -0.28229200

H 5.83446100 0.25257500 0.19483700

H 4.06467200 1.98440900 0.17984900

H 2.84248800 -2.80865800 -0.04776300

H 5.22440600 -2.14169200 0.08572600

H -3.09098600 2.59645600 -0.33851500

O 0.39445500 -2.39888200 -0.16231200

O 1.69892900 2.79380900 0.17822200

O -2.03063500 -1.78770400 -0.37412100

H -1.19879500 -2.36025600 -0.31464400

O -3.78275400 0.16495900 -1.17000800

H -4.33675500 -0.52264400 -0.75719000

O -3.19716400 0.58790700 1.05893500

O -4.78782000 -1.34016800 1.03551200

H -3.95992600 -1.81534500 1.23869300

48

TS18-19, E(SCF) = -1413.22382165, E(ZPE) = -1412.855985, E(G) = -1412.913671 a.u.

C -0.66790600 4.17568100 -0.70191000

C 0.75514500 3.71212200 -0.58021100

C -1.49656300 2.91931900 -0.55675400

C -0.59955400 1.75134200 -0.58287700

C 0.82056600 2.19529100 -0.77093900

H 1.17029200 2.03914300 -1.79969400

C -0.91128600 0.41932500 -0.33820000

C 1.81522000 1.53324200 0.20905800

O 1.71337000 4.40810000 -0.35313100

O -2.71319900 2.90753400 -0.50257600

C -2.20542800 -0.09514200 0.12370100

C -2.73424900 -1.23164400 -0.50082700

C -2.87284600 0.47631300 1.20258200

C -3.94664100 -1.74774100 -0.07870300

H -2.19527000 -1.69040400 -1.31999600

C -4.07518000 -0.05521900 1.64097500

H -2.44525100 1.33255000 1.70825900

C -4.61588400 -1.15892200 1.00318600

H -4.60350400 0.37203900 2.48384700

C 2.51960900 0.28211100 -0.25984400

C 3.45120900 -0.27217600 0.62832800

C 2.29739200 -0.32742900 -1.48257300

C 4.12972000 -1.42705300 0.29334300

H 3.62811500 0.21034500 1.58124500

C 2.99006800 -1.48825300 -1.82838300

H 1.58858400 0.08010500 -2.19229000

C 3.89728700 -2.03891000 -0.94957700

H 2.82447200 -1.97038700 -2.78352300

O 5.05069100 -2.06097000 1.07228300

O 4.57125500 -3.17934300 -1.28713600

H 5.16547300 -3.41703100 -0.55959500

O -5.79841900 -1.67678300 1.44204200

O -4.57708000 -2.83151200 -0.61653800

C -3.98073600 -3.42734500 -1.76457800

H -4.65089700 -4.22512800 -2.07245700

H -3.88251600 -2.69619900 -2.56953100

H -3.00186100 -3.84342000 -1.51890300

C 5.29645900 -1.51810600 2.36633100

H 5.70022100 -0.50684400 2.28962100

H 4.38039800 -1.51027100 2.95992600

H 6.02986100 -2.17167300 2.83045300

H 2.55584700 2.28027200 0.50119200

H -0.27687700 -0.31786200 -0.82009900

O 0.24288700 0.23821000 1.27352100

O 1.08571700 1.30384300 1.42541900

H -0.92725000 4.93719400 0.03355900

H -6.03860700 -2.43340700 0.88570900

H -0.82140100 4.59752100 -1.70155400

48

TS20-21, E(SCF) = -1413.22198342, E(ZPE) = -1412.853254, E(G) = -1412.911305 a.u.

C 0.24204300 3.69874500 -0.98503300

C 1.31246700 2.71308200 -0.57843200

C -1.06074700 2.95526300 -0.80939300

C -0.78055500 1.61594500 -0.12442200

C 0.69050300 1.33569200 -0.44849800

H 0.74090500 0.89421300 -1.45137900

C -1.67609000 0.50628100 -0.54717800

C 1.36791900 0.44021400 0.57796800

O 2.47311300 2.97250600 -0.37404800

O -2.15163600 3.34427200 -1.14034700

C -2.87468600 0.15517700 0.05909500

C -3.70965800 -0.80876300 -0.59605300

C -3.27341000 0.66900000 1.34435000

C -4.87304200 -1.21774800 -0.03210900

H -3.38693000 -1.18608400 -1.55758900

C -4.42752800 0.27523100 1.91897100

H -2.64317800 1.38839400 1.85062400

C -5.30259500 -0.69578600 1.28167300

H -4.74655000 0.65600000 2.88109000

C 2.55720300 -0.32726600 0.06934800

C 3.75042900 -0.26931700 0.79476800

C 2.48649300 -1.09914200 -1.08337700

C 4.86044700 -0.97385200 0.36055400

H 3.79849900 0.33513800 1.69141000

C 3.59693100 -1.81791200 -1.51285100

H 1.56804900 -1.15255300 -1.65449200

C 4.77875800 -1.75589000 -0.79929400

H 3.55883300 -2.42735300 -2.40709500

O 6.08010400 -0.98956300 0.97076200

O 5.86807800 -2.45948100 -1.23147900

H 6.60414800 -2.30101100 -0.62189600

O -6.35512900 -1.07675900 1.80056400

O -5.74032800 -2.09954500 -0.55721500

C -5.39981000 -2.67790800 -1.81560700

H -6.20852400 -3.36050100 -2.05958700

H -5.32118700 -1.90474900 -2.58231300

H -4.45856200 -3.22588600 -1.74101300

C 6.21629600 -0.24857600 2.17986200

H 6.03836400 0.81366500 2.00223700

H 5.52423900 -0.62152000 2.93733500

H 7.23982800 -0.39976000 2.51140000

H -0.86732000 1.82826000 0.94752600

H 1.65499900 1.03141400 1.44869500

H -1.51335400 0.15000000 -1.55963400

O -0.41789000 -0.98307600 0.17516800

O 0.35451300 -0.45243000 1.13573000

H 0.26837800 4.59891800 -0.36698400

H 0.37456200 4.00119900 -2.02745400

48

TS22-23, E(SCF) = -1413.21653929, E(ZPE) = -1412.846607, E(G) = -1412.906507 a.u.

C 0.05461500 3.06082600 -0.56402200

C 1.21579900 2.20473900 -0.41780600

C -1.04362500 2.34248700 -0.25127800

C -0.74018000 0.94487100 0.18236400

C 0.72406000 0.77517200 -0.21467900

H 0.75951300 0.30364100 -1.20405600

C -1.56672500 -0.16919500 -0.46390900

C 1.51979900 -0.09763800 0.76473700

O 2.39045400 2.54653800 -0.47416400

O -2.32115800 2.71438300 -0.23847400

C -2.95424500 -0.33690900 0.07544200

C -4.03374800 -0.38268700 -0.78175700

C -3.07832100 -0.60330500 1.51116000

C -5.29535500 -0.59153600 -0.24454700

H -3.89981500 -0.18786200 -1.83796700

C -4.26419800 -0.81374800 2.08227700

H -2.16799300 -0.61085700 2.09860400

C -5.47557700 -0.85492700 1.25373800

H -4.39206500 -0.99176500 3.14260100

C 2.84531600 -0.54975500 0.18382800

C 4.04271800 -0.07293900 0.71674400

C 2.87534500 -1.43913700 -0.88323100

C 5.25435000 -0.47420200 0.17467700

H 4.01871100 0.61927000 1.54870400

C 4.08911200 -1.85094200 -1.42239500

H 1.94677600 -1.81914200 -1.29402700

C 5.27447200 -1.36753800 -0.89996300

H 4.12742200 -2.54494300 -2.25280600

O 6.48685600 -0.07203400 0.60579300

O 6.47026400 -1.77541600 -1.43697700

H 7.18901400 -1.34439200 -0.95112900

O -6.57897100 -1.08612500 1.68074100

O -6.40871600 -0.56312500 -0.87614300

C -6.42549800 -0.32148900 -2.30380300

H -7.47494200 -0.33488100 -2.57902200

H -5.98065800 0.65221100 -2.50615000

H -5.88013600 -1.12013100 -2.80440700

C 6.53019800 0.86123100 1.68154100

H 6.01430100 1.78453800 1.41069800

H 6.08075000 0.43550300 2.57998600

H 7.58308100 1.06608700 1.85985100

H -0.83698500 0.89608200 1.27101000

H 1.70554400 0.50513100 1.66560900

H -1.62470900 0.04216500 -1.54334000

O -1.02198100 -1.41396700 -0.19187100

O 0.71793000 -1.18459000 1.14131000

H -2.42355500 3.63764200 -0.52009600

H 0.08845700 4.10612300 -0.84049200

48

TS24-27, E(SCF) = -1413.29585784, E(ZPE) = -1412.926325, E(G) = -1412.984307 a.u.

C -1.33740100 4.19408200 0.02561600

C -2.42404800 3.27282100 0.20834100

C -0.16549400 3.50338400 0.17486100

C -0.37842800 2.11353400 0.45817800

C -1.86231800 1.84888500 0.38960000

H -2.22852600 1.45281900 1.33888200

C 0.66733400 1.38495000 1.04731900

C -2.44759900 1.01545800 -0.77307100

O -3.62349000 3.52038800 0.19736500

O 1.08028000 3.98052100 0.13717700

C 1.48936100 -0.00368600 0.47083300

C 2.86700800 -0.07308100 0.88388100

C 1.11049900 -0.44074900 -0.84419600

C 3.81912600 -0.41945600 -0.01125100

H 3.09834800 0.19047800 1.90793600

C 2.05246700 -0.78589600 -1.74138100

H 0.06018200 -0.45842600 -1.09636800

C 3.46553400 -0.79035700 -1.40059300

H 1.79089000 -1.09324200 -2.74607500

C -2.27478900 -0.49049200 -0.68482100

C -2.25647100 -1.14857400 0.54766000

C -2.17455800 -1.23758100 -1.85197300

C -2.09350600 -2.52263900 0.60070600

H -2.34757700 -0.59454800 1.47065600

C -2.01481400 -2.61977900 -1.80096700

H -2.19647200 -0.74341200 -2.81367400

C -1.96766500 -3.26370300 -0.58183400

H -1.92508200 -3.20435500 -2.70819100

O -2.03568400 -3.26630700 1.74314600

O -1.80182400 -4.62167000 -0.52884400

H -1.78319200 -4.89585700 0.40026600

O 4.34133300 -1.09426500 -2.21312800

O 5.14003900 -0.48952900 0.23194700

C 5.57396100 -0.14724900 1.54653600

H 6.65417800 -0.25942200 1.54393800

H 5.30749200 0.88556000 1.77968500

H 5.13227500 -0.82255800 2.28192000

C -2.09453000 -2.56874800 2.98335000

H -3.04734700 -2.04622000 3.08731000

H -2.00576300 -3.32531000 3.75814700

H -1.26887700 -1.85861600 3.06436700

H -3.51966900 1.22719500 -0.75150700

H 1.44969600 1.95396300 1.53958500

O 0.51619600 0.07329900 1.48643900

O -2.00097300 1.53657500 -2.01462500

H 1.07502700 4.94178400 0.01443800

H -1.44072000 5.25294500 -0.16066300

H -1.05390200 1.35839100 -2.10722300

48

TS25-26, E(SCF) = -1413.31529586, E(ZPE) = -1412.944413, E(G) = -1413.000719 a.u.

C 1.74181500 2.86735900 -0.74242800

C 0.50951600 3.15006400 -0.03022800

C 1.61228400 1.69702400 -1.40688300

C 0.28924600 1.02650000 -1.19892800

C -0.42176100 2.04427100 -0.34034600

C 0.29867400 -0.22967600 -0.25818100

C -1.53861800 1.66968900 0.41066600

O 0.27576400 4.12660100 0.68207100

O 2.47840500 1.10615300 -2.22294900

C 1.61152700 -0.96746300 -0.20424800

C 2.65426500 -0.48303400 0.59241000

C 1.81612700 -2.07907300 -1.00678800

C 3.88727300 -1.11012900 0.56682100

H 2.48756700 0.38190100 1.22104800

C 3.05856700 -2.70739400 -1.03836400

H 1.00638400 -2.45753000 -1.61935800

C 4.09181400 -2.22346900 -0.26161900

H 3.23530000 -3.57398800 -1.66344700

C -2.55520600 0.72082700 -0.07815500

C -3.20546000 -0.11290500 0.84251700

C -2.88207100 0.67187300 -1.42597500

C -4.16997000 -0.99314700 0.39748900

H -2.92606200 -0.08506800 1.88864900

C -3.85960200 -0.21149800 -1.87014500

H -2.40160400 1.33829200 -2.13039000

C -4.49618100 -1.04293200 -0.97068400

H -4.13721500 -0.25800900 -2.91529200

O -4.86536200 -1.86673700 1.17260700

O -5.44517700 -1.91183100 -1.41075400

H -5.79443300 -2.40899200 -0.65531100

O 5.31426100 -2.84080900 -0.29101500

O 4.98088300 -0.73884500 1.29413400

C 4.86121200 0.42406900 2.10687600

H 5.83419400 0.56959400 2.56828700

H 4.60716300 1.29479600 1.49849400

H 4.10489900 0.27898600 2.88064500

C -4.60084000 -1.85461100 2.57314400

H -3.56161900 -2.12473500 2.76998500

H -4.81744400 -0.87138400 2.99513000

H -5.26375100 -2.59633500 3.00944000

H -0.16597100 0.80565000 -2.16472500

O -1.81769300 2.37034200 1.50473200

O -0.05709900 0.27900000 1.00592300

H -0.46990800 -0.91708900 -0.63059400

H 5.91542200 -2.36468200 0.30075300

H -2.68412800 2.12210600 1.86395900

H 3.29986200 1.61852500 -2.28603100

H 2.60383700 3.51888400 -0.75094400

48

TS28-29, E(SCF) = -1413.28842608, E(ZPE) = -1412.919201, E(G) = -1412.976854 a.u.

C 2.71612800 3.88153700 -0.06937800

C 3.21517600 2.52739500 0.05071500

C 1.40380700 3.83560300 -0.44712800

C 0.97394400 2.48329700 -0.66576400

C 2.09463200 1.55780800 -0.29990300

H 2.41806600 0.87300700 -1.08324500

C -0.32693800 2.04647300 -0.72077800

C 1.52089800 0.82679900 0.96131000

O 4.33233400 2.17763100 0.40781000

O 0.54698400 4.83902000 -0.61964200

C -1.83083800 0.25868600 -1.00476800

C -2.66748600 0.65413200 0.03335300

C -2.16369200 -0.76286100 -1.86785200

C -3.88970600 0.01385700 0.18397000

H -2.36081500 1.41927300 0.73128000

C -3.38701200 -1.40320200 -1.70015300

H -1.48156400 -1.05185300 -2.65584900

C -4.25023400 -1.02252500 -0.69017200

H -3.67821800 -2.20902100 -2.36383200

C 1.38025000 -0.67217700 0.76211200

C 2.49386600 -1.39910500 0.32747400

C 0.20041400 -1.33739600 1.04739000

C 2.40855900 -2.77036000 0.16544400

H 3.42346000 -0.88274200 0.11838600

C 0.10993700 -2.71962700 0.87915300

H -0.66257700 -0.78339400 1.39136700

C 1.20413600 -3.43446200 0.44045700

H -0.81129100 -3.24844300 1.09197100

O 3.42181900 -3.58388100 -0.25544200

O 1.11618300 -4.79400900 0.27577200

H 1.97653700 -5.12464700 -0.02197200

O -5.46629300 -1.61596800 -0.47836500

O -4.79141500 0.30801600 1.15708700

C -4.46043000 1.35547500 2.06390500

H -5.30527800 1.43805600 2.74209000

H -3.55916500 1.10951500 2.62941300

H -4.32088700 2.29914400 1.53269700

C 4.67427000 -2.97006100 -0.54130900

H 5.07625300 -2.48179300 0.34869300

H 5.33853500 -3.77202700 -0.85128500

H 4.57235800 -2.24254900 -1.34912700

H 2.22039700 0.99052800 1.79147400

H -1.16657300 2.72423900 -0.59772900

O -0.57454800 0.83127700 -1.19332400

O 0.30924500 1.47396800 1.27169900

H 3.29140900 4.77311900 0.13428200

H 0.96685000 5.68975200 -0.41969000

H -5.61005100 -2.30431300 -1.14217300

50

TS30-31, E(SCF) = -1414.42122771, E(ZPE) = -1414.034880, E(G) = -1414.097501 a.u.

C -0.11128000 -0.41933000 -0.02217900

C -1.43012200 -1.00702600 -0.15502900

C 1.02246900 -1.18684900 -0.04032900

C 2.30484000 -0.57018700 0.07773600

H 2.34175700 0.50123500 0.21736300

C -2.56700500 -0.08798900 -0.10700900

H -2.33031800 0.96456500 -0.01326400

C 3.57142900 -1.33271800 0.06881700

H 3.49910000 -2.31737000 -0.39253100

C -3.83082500 -0.53617200 -0.16257100

H -3.98534400 -1.60944000 -0.24252900

O -1.56641100 -2.24086500 -0.29129500

O 1.00185600 -2.51845300 -0.17769100

C 4.81831700 -0.60182100 -0.32001000

C 5.15280800 0.59135800 0.32861700

C 5.63742000 -1.10609100 -1.31612600

C 6.29814300 1.27173000 -0.03788700

H 4.51564800 0.97395900 1.11600400

C 6.79267500 -0.42020200 -1.68676400

H 5.37989400 -2.03531600 -1.80988500

C 7.12311800 0.75972000 -1.05313300

H 7.44591400 -0.79917700 -2.46255900

C -5.04329900 0.27084300 -0.11928700

C -6.27954300 -0.39614500 -0.11734100

C -5.02433700 1.66681600 -0.07993300

C -7.46081300 0.31953900 -0.07183400

H -6.29494700 -1.47791200 -0.14907900

C -6.20840100 2.38475500 -0.03696000

H -4.08591000 2.20536400 -0.08524300

C -7.42391000 1.72095500 -0.03305000

H -6.20676500 3.46705000 -0.00951500

O -8.71499700 -0.21427100 -0.06171700

O -8.58048400 2.43837800 0.00759900

H -9.33326800 1.82760300 -0.00081800

O 8.25822300 1.43120200 -1.41375200

H 8.33027000 2.23268200 -0.87400300

O 6.73532400 2.44197900 0.50675500

C 5.92501700 3.03610500 1.51657400

H 6.42482300 3.95917800 1.79695600

H 4.92874700 3.25718400 1.12820300

H 5.84894200 2.37893600 2.38495800

C -8.82166200 -1.63439800 -0.09665700

H -8.37824000 -2.03131100 -1.01183000

H -8.33708000 -2.07842500 0.77505300

H -9.88520000 -1.85533200 -0.07790100

H -0.01461000 0.65001300 0.10075700

H 0.04918700 -2.77947900 -0.25314600

O 3.28987400 -1.36231600 1.45025200

O 4.50275500 -2.30485300 2.28204700

H 4.00804400 -3.13775800 2.23155600

50

TS35-36, E(SCF) = -1414.44781095, E(ZPE) = -1414.060064, E(G) = -1414.122728 a.u.

C 0.03869200 -3.63447600 -0.65618700

C 1.20183800 -2.91456100 -0.00914100

C -1.26215700 -3.12301200 0.00464400

C -1.72047800 -1.80222300 -0.42619200

H -1.15939400 -1.31700300 -1.21645200

C 1.73179800 -1.69181600 -0.50383900

H 1.40839300 -1.39906200 -1.49414500

C -2.79998600 -1.23999700 0.14022700

H -3.30058600 -1.79612000 0.92930600

C 2.60434000 -0.96491400 0.23186600

H 2.87821600 -1.34168000 1.21353300

O 1.50415700 -3.30295600 1.19573900

O -1.82340500 -3.81570500 0.83477200

C -3.39381800 0.04896600 -0.18341900

C -4.51786600 0.45166100 0.55679000

C -2.90622400 0.88292400 -1.19167900

C -5.13281000 1.65952200 0.29193900

H -4.89377200 -0.19780500 1.33658100

C -3.52469100 2.09369500 -1.45946400

H -2.04173600 0.59242500 -1.77497000

C -4.63446200 2.48402900 -0.72732800

H -3.15946300 2.74931700 -2.23973000

C 3.22698600 0.28173600 -0.14534900

C 4.11226500 0.87347800 0.77578800

C 2.99616400 0.90622800 -1.37598700

C 4.74694800 2.05706700 0.46593200

H 4.28698300 0.38573100 1.72610400

C 3.63353600 2.09345500 -1.68632700

H 2.31781300 0.46785300 -2.09611300

C 4.50621400 2.67058800 -0.77531400

H 3.46789700 2.58942900 -2.63404100

O 5.62077700 2.72855700 1.26418500

O 5.12630700 3.83358000 -1.08851700

H 5.69693500 4.09880300 -0.34993800

O -5.23722100 3.67299200 -1.00186300

H -5.99723100 3.78446100 -0.41044900

O -6.22292000 2.15800800 0.94030400

C -6.76675900 1.37686300 2.00046300

H -7.60737400 1.94465300 2.38966500

H -7.11462700 0.41145400 1.62821800

H -6.02449500 1.22817100 2.78694000

C 5.90202200 2.16406500 2.54232200

H 4.99214300 2.10215800 3.14231300

H 6.34681500 1.17308800 2.43433100

H 6.61120400 2.83671600 3.01636000

H 0.01218900 -3.36364000 -1.71729200

H 2.63999300 -4.99354100 -0.35638300

O 0.23208000 -4.97949200 -0.41864800

O 2.21345300 -4.52250600 -1.08693100

H 1.08496700 -4.17489500 1.35536000

28

TS5-6, E(SCF) = -989.644480909, E(ZPE) = -989.453897, E(G) = -989.498174 a.u.

C 4.70709200 0.12347900 0.19308900

C 3.67589900 1.04825000 0.15956800

C 2.35781500 0.61408800 0.04075000

C 2.07269500 -0.75125500 -0.03937000

C 3.11335200 -1.67350500 0.00176900

C 4.42472800 -1.23724800 0.11543500

C 1.26226100 1.60830100 0.01266400

C 0.66845800 -1.24473000 -0.16758500

C -0.39960100 -0.25080200 -0.25822400

C -0.13889800 1.11042900 -0.14984400

C -1.15312400 2.09796100 -0.20602800

H -0.86201600 3.13801800 -0.16224000

C -2.46563400 1.75244500 -0.28157400

C -2.88249300 0.33551400 -0.20656800

C -1.77008600 -0.67418700 -0.52866200

H 5.73173200 0.46101300 0.28179000

H 3.88227800 2.10870500 0.22329900

H 2.88844300 -2.73008800 -0.05799400

H 5.23157800 -1.95863100 0.14357100

H -3.26212300 2.48548100 -0.29911500

O 0.45513400 -2.45249200 -0.17564100

O 1.48687200 2.80246200 0.11554400

O -2.10158700 -1.76173800 -0.97904400

O -4.02966900 0.06450700 -0.95714500

H -4.09430400 -0.90132900 -1.03152800

O -3.11545100 0.36070600 1.16724600

O -3.64413700 -1.28717500 1.68283400

H -2.75965700 -1.56576500 1.95933900

28

TS6-7, E(SCF) = -989.663002646, E(ZPE) = -989.473234, E(G) = -989.517953 a.u.

C 4.76802100 -0.02757500 0.18956900

C 3.77447600 0.93926500 0.14441400

C 2.44190300 0.55564500 0.04257900

C 2.10490000 -0.79980500 -0.01456100

C 3.10406600 -1.76387400 0.03813500

C 4.43307700 -1.37648800 0.13601600

C 1.38554900 1.59194400 -0.00327900

C 0.68264000 -1.22662900 -0.10771800

C -0.34876100 -0.16669700 -0.22660100

C -0.04294500 1.14513500 -0.13461000

C -1.06205800 2.19091400 -0.18429900

H -0.72446400 3.21731800 -0.23037800

C -2.35866300 1.88981900 -0.14525300

C -2.83904000 0.46230500 -0.02496300

C -1.76488000 -0.53191900 -0.52232100

H 5.80572300 0.27018800 0.26774500

H 4.02372400 1.99153000 0.19009100

H 2.83629000 -2.81168500 -0.00207500

H 5.21103400 -2.12844700 0.17303800

H -3.13428400 2.64632800 -0.13154000

O 0.37498500 -2.40218100 -0.05538000

O 1.64094800 2.77717400 0.05385500

O -2.06305900 -1.53967800 -1.11616700

O -4.04798200 0.26931600 -0.67046300

H -4.37711100 -0.57160000 -0.13242800

O -2.96336200 0.24824200 1.34983800

O -4.39300000 -1.56603700 0.94059500

H -3.69886500 -2.20056400 0.72466500

2

OH-RAD_PCM, E(SCF) = -75.7309936119, E(ZPE) = -75.722441, E(G) = -75.739363 a.u.

O 0.00000000 0.00000000 0.10806000

H 0.00000000 0.00000000 -0.86448000

3

OOH-RAD_PCM, E(SCF) = -150.896225870, E(ZPE) = -150.881916, E(G) = -150.904065 a.u.

O 0.05521400 0.70928700 0.00000000

O 0.05521400 -0.60017000 0.00000000

H -0.88342700 -0.87293300 -0.00000000

3

H2O_PCM, E(SCF) = -76.4275457413, E(ZPE) = -76.406131, E(G) = -76.423770 a.u.

O 0.00000000 0.00000000 0.11783700

H 0.00000000 0.75762900 -0.47134900

H 0.00000000 -0.75762900 -0.47134900

4

H2O2_PCM, E(SCF) = -151.541115024, E(ZPE) = -151.514399, E(G) = -151.536010 a.u.

O 0.00000000 0.71317500 -0.06422800

H 0.74927300 0.91082300 0.51382200

O 0.00000000 -0.71317500 -0.06422800

H -0.74927300 -0.91082300 0.51382200

2

O2_M3_PCM, E(SCF) = -150.307170973, E(ZPE) = -150.303305, E(G) = -150.323255 a.u.

O 0.00000000 0.00000000 0.59820800

O 0.00000000 0.00000000 -0.59820800

2

SUPEROXIDE-RAD_PCM, E(SCF) = -150.427313515, E(ZPE) = -150.424452, E(G) = -150.444219 a.u.

O 0.00000000 0.00000000 0.66413300

O 0.00000000 0.00000000 -0.66413300
